# Host-trailing satellite flight behaviour is associated with greater investment in peripheral visual sensory system in miltogrammine flies

**DOI:** 10.1038/s41598-022-06704-8

**Published:** 2022-02-17

**Authors:** Carlo Polidori, Marcin Piwczynski, Federico Ronchetti, Nikolas P. Johnston, Krzysztof Szpila

**Affiliations:** 1 Dipartimento di Scienze e Politiche Ambientali, Università Degli Studi di Milano, via Celoria 26, 20133 Milan, Italy; 2 Department of Ecology and Biogeography, Nicolaus Copernicus University, Lwowska 1, 87-100 Toruń, Poland; 3 Department of Animal Ecology and Tropical Biology, University of Wuerzburg, Hubland Nord, 97074 Würzburg, Germany; 4 School of Life Sciences, University of Technology Sydney, 15 Broadway, Ultimo, NSW 2007 Australia

**Keywords:** Behavioural ecology, Evolutionary ecology, Entomology

## Abstract

Insect sensory systems are the subjects of different selective pressures that shape their morphology. In many species of the flesh fly subfamily Miltogramminae (Diptera: Sarcophagidae) that are kleptoparasitic on bees and wasps, females perch on objects close to the host nests and, once a returning host is detected, they follow it in flight at a fixed distance behind until reaching the nest. We hypothesized that such satellite (SAT) flight behaviour, which implies a finely coordinated trailing flight, is associated with an improved visual system, compared to species adopting other, non-satellite (NON-SAT) strategies. After looking at body size and common ancestry, we found that SAT species have a greater number of ommatidia and a greater eye surface area when compared to NON-SAT species. Ommatidium area is only affected by body size, suggesting that selection changes disproportionately (relative to body size variation) the number of ommatidia and as a consequence the eye area, instead of ommatidium size. SAT species also tend to have larger ocelli, but their role in host-finding was less clear. This suggests that SAT species may have a higher visual acuity by increasing ommatidia number, as well as better stability during flight and motion perception through larger ocelli. Interestingly, antennal length was significantly reduced in SAT species, and ommatidia number negatively correlated with antennal length. While this finding does not imply a selection pressure of improved antennal sensory system in species adopting NON-SAT strategies, it suggests an inverse resource (i.e. a single imaginal disc) allocation between eyes and antennae in this fly subfamily.

## Introduction

The insect sensory system is the part of the nervous system which process both internal and external stimuli, through signal transfer from sensory receptors to the brain. Such receptors, depending to the type of energy they transduce, can be classified into different categories, e.g. light and/or visual detectors, mechanoreceptors or chemoreceptors^1–3^. Because of their fundamental role in a wide range of activities, insect sensory systems are subject to strong selective pressures^2,4^. Hence, it is not surprising that a link between sensory system and different life-history or behavioural traits is expected. For example, resource-finding activities (e.g. related to mating and foraging) in insects extensively rely on both visual and olfactory cues emitted by the resources themselves or by habitat components related to the presence of resources^5–7^. Thus, besides brain morphology, morpho-anatomical traits related to both the peripheral visual (eyes and ocelli) and the olfactory sensory systems (essentially located in the antennae) could have evolved to optimise the strategies adopted to find resources^8–11^. Because the visual and olfactory system evolve in response to different types of stimuli, an inverse allocation between vision and olfaction was also detected in some insects spanning diverse groups such as flies, moths, bees and ants, i.e. after taking into account body size and phylogenetic relationships, visual system- and olfactory system-related traits are inversely correlated^12–17^. Furthermore, certain activities depend on correct sensing of mechanical stimuli, which are also detected by specialised sensilla mainly located on antennae^18–20^.

In this study, we focus on the visual system of Miltogramminae (Diptera: Sarcophagidae). The peripheral visual system in insects is composed of two units: the compound eyes and the ocelli. Compound eyes consist of a repetitive structure, the ommatidium, each usually containing a fixed number of neuronal photoreceptors, pigment cells and lens-secreting cone cells^21^. The number and size of ommatidia, and then ultimately eye size, determines the ability to capture light and the image resolution (acuity)^22–24^. In contrast to the multi-lensed compound eyes, the dorsal ocelli of insects are simple lens eyes which externally consist of a single, usually round or oval aperture lens while internally hundreds of photoreceptors converge into a small suite of neurons targeted to neuropils^25,26^. Although these simple eyes cannot capture forms or can capture forms to a very limited extent (i.e. they essentially lack optical resolving power), they are better than compound eyes at capturing light^11,25^. A link between visual system and resource-finding behaviour in insects has been highlighted in several studies. For example, larger eyes and larger ocelli were observed in different insect species with crepuscular or nocturnal foraging^8,11,24,27^. In some ant species, similar-sized individuals with different modes of locomotion (reproductive alates vs. workers) have different visual system morphology, suggesting a non-allometric relationship driven by visual processing needs associated with different behaviours^28,29^.

The Miltogramminae are an interesting model to test the hypothesis that resource-finding strategies drive the evolution of visual system. In this taxon there are three main groups of species that could be categorised in relation to the behaviour they utilise to find resources^30,31^. The first group includes the necrophagous species (e.g. *Phylloteles* spp.), which use various types of animal carrion to feed their brood. The kleptoparasitic species, which mainly attack nests of wasps and bees (Hymenoptera: Aculeata), can be then divided into the second and third groups, based on their host-finding strategies. Species from the second group wait on perching sites, close to host nest entrances, for a nest-returning host female, then follow it in flight at a fixed distance behind (“satellite flies”), ultimately sneaking into the nest (e.g. *Senotainia* spp., *Pterella* spp.). The third group is composed of species which either patrol the host nesting site and enter the host nests (“hole searchers” (e.g. *Metopia* spp.)) or enter the host nest after having detected the female host entering (“stalkers and lurkers” (e.g. *Taxigramma* spp.)). Stalkers and lurkers differentiate from satellite flies in that, despite both relying on host presence to identify host nests, the former do not engage in the complex host-trailing flights typical of the latter. The larvae of parasitic miltogrammine are primarily kleptoparasites and devour the host larval food, but may also initially destroy the host eggs and larvae^32–34^; one species, *Senotainia tricuspis* (Meigen), attacks adult hymenopterans^35,36^. Host species span many families of wasps (Crabronidae, Pompilidae, Sphecidae, Vespidae) and bees (Andrenidae, Apidae, Colletidae, Halictidae) representing various life strategies, including solitary, social, ground-nesting and aerial-nesting species^31,37–41^. Several species of the early evolutionary branch of Miltogramminae invade termite or ant nests^42^.

Because of the unique, particularly elaborate host-trailing satellite behaviour, whose precision likely strongly depends on vision, we here hypothesized that satellite (SAT) fly species possess an improved visual system. On the other hand, species that do not perform satellite flights (NON-SAT: necrophagous, hole searchers and stalkers and lurkers) would not need such an improved visual system. Additionally, we measured antennal size of SAT and NON-SAT species. Because the exact function of the different types of sensilla is unknown in Miltogramminae, we did not attempt to associate antennal size with chemical or mechanical sensitivity. However, independently from the relative involvement of antennae in mechano- and olfactory reception, there it could be a trade-off during development of antennae and eyes, which are formed in holometabolous insects from the same imaginal disc^43^. Hence, we tested if an inverse resource (i.e. the imaginal disc) allocation between these two main sensory organs occurs across Miltogramminae species, opening to new hypotheses to test in the future on how the found patterns may potentially relate with differential investment in different senses in these flies.

## Materials and methods

### Study species and origin of sample

We analysed females (the only sex searching/pursuing hosts) of a total of 18 species of Miltogramminae which belong to 12 genera: *Amobia signata* (Meigen), *Apodacra seriemaculata* Macquart, *Craticulina tabaniformis* (Fabricius), *Eumacronychia persolla* Reinhard, *Metopia argyrocephala* (Meigen), *Miltogramma germari* Meigen, *Miltogramma punctata* Meigen, *Miltogramma turanica* Rohdendorf, *Phrosinella fedtshenkoi* Rohdendorf, *Phrosinella kocaki* Verves & Khrokalo, *Phylloteles pictipennis* Loew, *Pterella melanura* (Meigen), *Senotainia albifrons* (Rondani), *Senotainia conica* (Fallén), *Senotainia tricuspis* (Meigen), *Sphenometopa claripennis* (Villeneuve), *Taxigramma heteroneura* (Meigen), *Taxigramma stictica* (Meigen). Ten of these species were SAT species and the other eight species were NON-SAT species (Fig. 2A). Among them, two were necrophagous while six were, similarly to SAT species, associated with aculeate hymenopterans but either hole searchers (three species) or stalkers and lurkers (three species) (Fig. 2A). Information about the resource-finding strategy of each species was retrieved from relevant publications (Supplementary Table S1). The studied individuals were collected using a hand net at different locations in Europe, Middle East, North Africa and North America (see Supplementary Table S2 for details). Upon collection, specimens were killed in fumes of ethyl acetate, stored dry on entomological pins and subsequently identified to species by one of the co-authors (KS). From 4 to 7 females *per* species (median = 7) were used in our morphological study (Supplementary Table S1).

### Scanning Electron Microscopy (SEM)

The entire pinned individual of each studied female was subjected to scanning electron microscopy (SEM). The head was photographed anteriorly and laterally, in order to obtain morphometric data from head, eye and ocelli. Pictures of antennae and sensilla were also taken at higher magnification to study details of their morphology.

SEM images were obtained using the Inspect Scanning Electron Microscope from the FEI Company (Oregon-USA) located at Museo Nacional de Ciencias Naturales (MNCN-CSIC) (Madrid, Spain). We operated at low-vacuum mode (resolution: 3.0 nm at 30 kV (secondary electrons, SE), 4.0 nm at 30 kV (Backscattered electrons, BSEs), and < 12 nm at 3 kV (SE)). These parameters allowed inspection at a high resolution and supported the analysis of non-conductive hydrated samples in their original condition with both the large field detector (LFD), (close to the sample and thus avoiding loss of electrons) and the backscatter detector (backscatter electron detector, BSED). The accelerating voltage was 26 kV; the vacuum was 0.40–0.50 torr; and the working distance was 10 mm.

### Morphological variables

From the SEM pictures we obtained a number of morphological quantitative variables. Researchers who quantified these variables (see below) were blind with respect to species identity and host-finding strategy.

**Figure 1:**
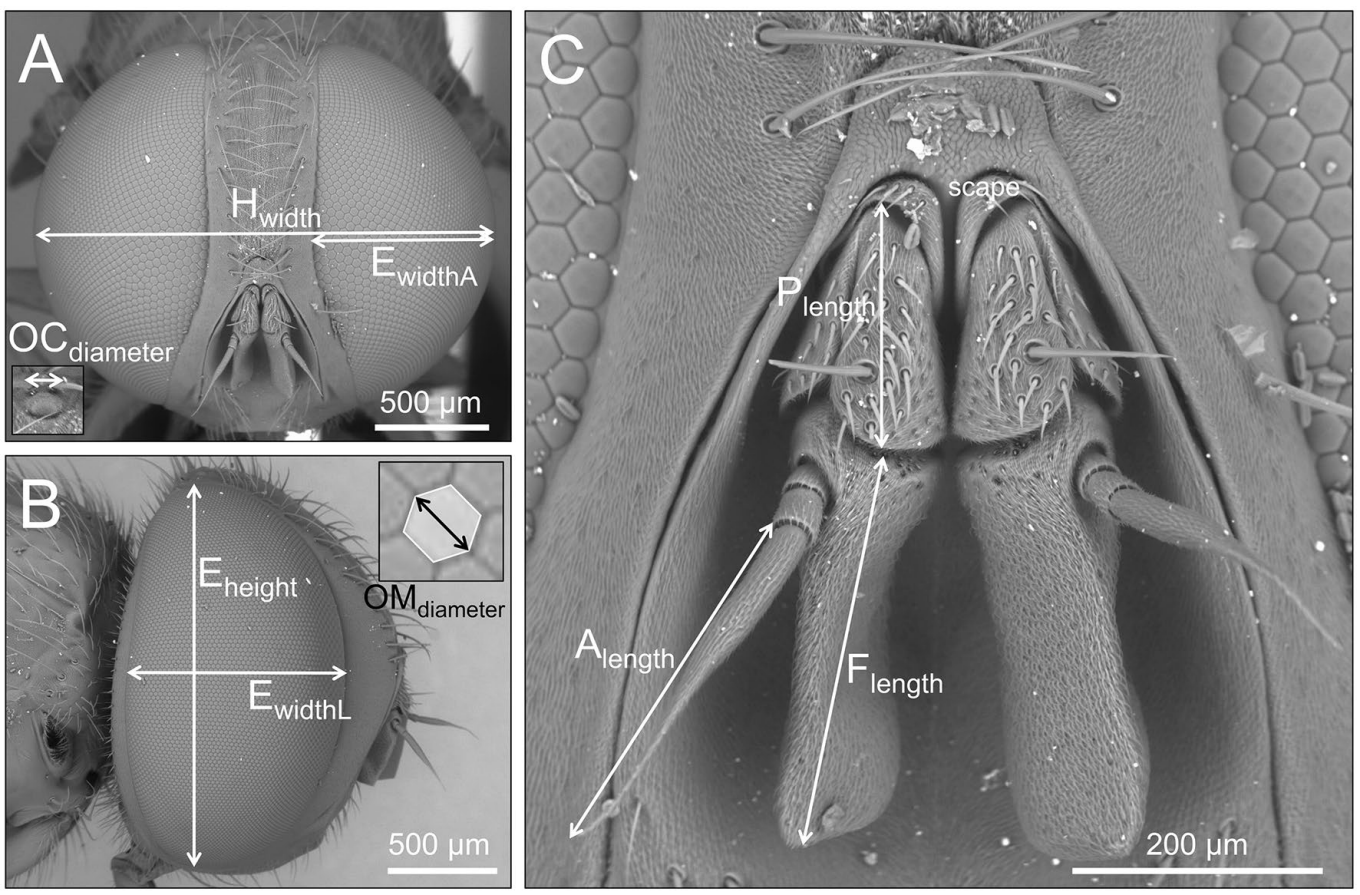
SEM pictures of *Miltogramma turanica* showing the measurements and counts obtained for the comparative morphological analyses of our studied miltogrammine species. (**A**) head in frontal view; (**B**) head in lateral view; C, antennae in frontal view. Abbreviations: H_width_ = head width, E_widthA_ = eye width in anterior view, E_height_ = eye height, E_widthL_ = eye width in lateral view, OM_diameter_ = 2 × hexagon radius, P_length_ = pedicel length, A_length_ = arista length, F_length_ = funiculus length.

The head width (H_width_), as the maximum distance between the outer margins of the eyes, was measured and used in the analysis as a proxy for body mass (Fig. 1). In brachyceran Diptera (which include the Sarcophagidae), a strong allometric relationship between head width and dry body mass (body mass = 0.655 × H_width_^2.526^, R^2^ = 0.93) was found ^44^. Moreover, the head width as a proxy of body mass is useful for practical reasons, since the head capsule is usually less susceptible to damage and deformity than other body parts ^45^.

With regards to the visual system, we analysed both the compound eyes and the ocelli. For each individual, either left or right eye was randomly chosen. We measured three linear variables related to eye size: the eye width (taken parallel to sagittal body axis, in lateral view, E_widthL_), the eye height (taken perpendicular to longitudinal body axis, in lateral view, E_height_) and eye width in anterior view (taken perpendicular to sagittal plane of the body, E_widthA_) (Fig. 1). We then used these three measurements to estimate the total eye area (E_area_), i.e. by approximating the eye to a spherical shell. Basically, if the eye height and eye width would have the same value (L), the eye would be a perfect spherical shell with eye height as height of the shell (H). The area of this spherical shell is by definition: 2π × H × r, where r is the radius of the sphere from which the spherical shell is cut, i.e. r = (L)^2^ + H^2^)/2H). Since the fly’s eye has different eye height and eye width, by averaging their values we will have the parameter L to be used in the equation above to calculate the eye area, which from our data is thus as follows:

$${\text{E}}_{{{\text{area}}}} = { 2}\pi \times {\text{E}}_{{{\text{widthA}}}} \times {{\left( {\left( {\left( {{\text{E}}_{{{\text{height}}}} + {\text{E}}_{{{\text{widthL}}}} } \right)/{2}} \right)^{{2}} + {\text{E}}_{{{\text{widthA}}}}^{{2}} } \right)} \mathord{\left/ {\vphantom {{\left( {\left( {\left( {{\text{E}}_{{{\text{height}}}} + {\text{E}}_{{{\text{widthL}}}} } \right)/{2}} \right)^{{2}} + {\text{E}}_{{{\text{widthA}}}}^{{2}} } \right)} {\left. {\left( {{2} \times {\text{ E}}_{{{\text{widthA}}}} } \right)} \right)}}} \right. \kern-\nulldelimiterspace} {\left. {\left( {{2} \times {\text{ E}}_{{{\text{widthA}}}} } \right)} \right)}}$$

We calculated the ommatidia size by measuring the radius (distance between two apex (diameter) divided by 2) of the hexagon (their typical shape) and by using it to calculate the area (OM_area_) following the formula: OM_area_ = 2.598 × radius^2^. We used the mean value of the OM_area_ across 10 ommatidia randomly chosen in the central part of one eye of each individual. We obtained the total number of ommatidia (OM_number_) by dividing the eye area by the mean ommatidia area. We used ocellar diameter (taken parallel to longitudinal body axis, in lateral view, OC_diameter_) as an estimate of their size (Fig. 1). Finally, we calculated the interommatidial angle, i.e. the inverse of the angle subtended between the optical axes of neighboring ommatidia, by using the general equation provided by ^22^: ∆γ = (23.818/OM_number_)^1/2^. Such parameter anatomically defines visual acuity; we here considered the global interommatidial angle (i.e. the average of all local ones from the various regions of the compound eye) ^46^.

Sarcophagid flies bear a pair of aristate antenna located between the compound eyes (Fig. 1). In this study we essentially follow the terminology given in Stuckenberg ^47^ (for antennal segments). The antenna is divided into three segments: a proximal scape, a pedicel, and a distal flagellum. The flagellum is composed of a three-segment arista (A) and a funiculus (F) (Fig. 1). We measured the following traits related to the antennae: funiculus length (F_length_), pedicel length (P_length_) and arista length (A_length_). We also determined the total antennal length (ANT_length_) by summing the three previously cited variables. The scape was not considered in this study, given that it represents a small portion of the whole antenna. At last, we give a preliminary overview of the different sensillar types by describing their external morphology and comparing it with that of sensilla described in other sarcophagid flies.

All counts and measures were taken on the SEM pictures with the software ImageJ (NIH, USA). The final data analysis was performed on the species mean values of the following 10 morphological variables: H_width_, E_area_, OM_area_, OM_number_, OC_diameter_, ∆γ, F_length_, P_length_, A_length_ and ANT_length_. The morphometric data for each individual are given in the Supporting information (Supplementary Table S2).

### Phylogenetic reconstruction

The phylogenetic tree used for the comparative analyses was obtained from a larger genomic project concerning phylogenetic relationships within the subfamily Miltogrammine, the results of which will be the topic of a separate publication. Briefly, total genomic DNA for 114 species, including the 18 used in this study, were extracted from the thorax, legs and abdomen of 95% ethanol-preserved specimens using a DNeasy Blood & Tissue kit (Qiagen, CA, USA). Anchored Hybrid Enrichment (AHE) libraries were then prepared following protocol established by Lemmon et al*.* ^48^, with modifications specific to Diptera based on protocols described in detail elsewhere ^49–52^. DNA libraries were subsequently enriched with an Agilent Custom SureSelect kit (Agilent Technologies, CA, USA), with probes designed specifically for Diptera (Young et al*.* 2016) targeting 559 loci (specific loci sequences available as Supplementary Material in Young et al*.* 2016). Enriched DNA libraries were then pooled and sequenced as single reads (100 bp) on an Illumina HiSeq 2500 platform at the NCSU Genomics Sciences Laboratory (Raleigh, NC).

Orthology prediction and sequence assembly followed the bioinformatic pipeline established by the 1KITE consortium (https://www.1kite.org) with additional modifications and quality control steps adapted from Misof et al*.* ^53^ and Buenaventura et al*.* ^51^. The resultant nucleotide sequence alignment was analysed using a maximum likelihood (ML) approach as implemented in IQ-TREE ^54^. All possible substitution models were tested in ModelFinder implemented within IQ-TREE ^54^ and the model with the highest corrected Akaike Information Criteron (AICc) (GTR + FreeRate model with 10 categories and empirical base frequencies ‘ + R10 + F’) was chosen for the final analysis. Node support for this phylogenetic tree was estimated using 10,000 ultrafast bootstrap replications. The resulted phylogenetic tree was rooted and subsequently pruned to consist of only the 18 species used in the comparative analyses. All nodes in this tree had the maximum bootstrap support providing a strong phylogenetic hypothesis for comparative study (see “Results”). The same topology of the phylogenetic tree for subfamily Miltogramminae was obtained by Buenaventura et al. ^51^, while Yan et al. ^55^ obtained a slightly different topology (see “Discussion”).

Since comparative methods based on an Ornstein–Uhlenbeck process require a dated tree, we made the tree ultrametric by dating it using a penalised likelihood method using the chronos function in the R package ape ^56^. We fixed the root to relative age 1, because there is no known fossil record for this group of flies that would serve as a suitable calibration point. Next, we mapped host-finding strategy on the tree using the maximum parsimony method in Mesquite version 3.61 ^57^. We obtained two maximum parsimony reconstructions, which were then used for the comparative analyses.

### Comparative approach

We used a comparative method designed to model adaptive evolution, following Hansen ^58^, Hansen et al. ^59^ and Labra et al. ^60^ as implemented in the R package SLOUCH (https://kopperud.github.io/slouch/) to study adaptive evolution of visual sensory system and size of antenna in Miltogramminae. In SLOUCH, the adaptive evolution of trait is modeled as an Ornstein–Uhlenbeck stochastic process expressed in the following stochastic differential equation:

$${\text{dy }} = \, - \alpha \left( {{\text{y }}{-} \, \theta } \right){\text{dt }} + \, \sigma {\text{dB}}$$

in which dy is the change in trait y (visual sensory and antenna size traits in our case) over time interval dt, α is a parameter measuring the strength of the pull towards an optimum θ, i.e. rate of adaptation, and σdB is a white-noise process. This model contains two components: deterministic, i.e. tendency to evolve toward an optimum and stochastic, i.e. evolutionary changes due to noise generated by secondary selection pressures, genetic drift, as well as other unmeasured variables affecting the evolutionary process. If α = 0 then the tendency to evolve toward an optimum disappears and trait evolution occurs according to Brownian motion. The optimum, here, also called primary optimum, is defined as average optimum reached by a number of species evolving in the same niche for an amount of time necessary to eradicate all ancestral constraints ^61^. In SLOUCH, primary optima can be either fixed by mapping niches onto a phylogeny (NON-SAT vs. SAT “niches” in our case) or can be modeled as a function of a randomly evolving predictor variable (head width, as a proxy for body mass, in our case). Several important parameters are returned by the method. The relative effects of phylogenetic inertia (resistance of adaptation) are described by a half-life t_1/2_ = ln(2)/α, interpreted as the average time necessary for a species to evolve halfway from an ancestral state toward a new optimum ^58^. A short half-life relative to phylogenetic tree length indicates rapid adaptation toward the optimum while a long half-life slow adaptation with strong influence of ancestral states. In the case where primary optimum is modeled as a function of a random predictor, the method returns an estimate of parameters of two kind of regressions, optimal and evolutionary regression. Optimal regression is a regression of primary optimum on the predictor variable and is interpreted as a relationship free of ancestral influence. Alternatively, evolutionary regression includes a phylogenetic correction factor due to inertia making it shallower than an optimal regression unless adaption is instantaneous.

We assumed that the direction of evolution of the visual system and size of antenna in miltogrammines is influenced by two factors: allometric relationship with body size (approximated by head width in our case) and SAT vs NON-SAT behavior. Therefore, for each trait we tested four models:

#### Model without predictor variables to estimate the overall phylogenetic effect

This effect can be due to phylogenetic inertia. i.e. a resistance in the evolution towards the optimal state or phylogenetic effect in the environment to which the species are adapting. The difference between half-lives estimated for models with and without predictor variables can inform us if the overall phylogenetic effect is due to inertia or due to environment. For example, if half-lives estimated without predictor variables were very long while with predictor variables were very short it could mean strong phylogenetic structure in the environment and a low level of phylogenetic inertia.

#### Model with body size (head width) as a continuous predictor variable evolving according to Brownian motion

Body size affects sizes of almost all morphological traits due to allometric relationships. As such, body size is often viewed as a strong constraint on the rate and direction of evolution. This model tested if changes in body size causes a strong response in visual system and antenna size.

#### Model with host-finding behavior as a fixed predictor

In this model, we tested whether there is a systematic effect of host-finding behavior (SAT vs NON-SAT) on the analysed traits of visual system and antenna.

#### Model with host-finding behavior and body size (head width) as predictors

We tested here the joint effect of host-finding behaviour and body size on the analysed traits of visual system and antenna. For example, one may expect of systematic changes in mean values between SAT and NON-SAT species in studied traits with allometric relationships preserved.

The models were performed for all the morphological variables listed above, except on ∆γ, which being essentially a ratio between a constant value and OM_number_ will follow an inverse pattern to that of OM_number_.

Lastly, to test if the different analysed traits related to visual system and antenna are inversely correlated in Miltogramminae, we performed a series of linear correlation Pearson tests between pairs of visual and antennal traits, using their values divided by head width (× 100) to account for differences in body size. Keesey et al. ^12^ also used the measurements divided by body size in their study on *Drosophila*, though they use multiple regressions to select the visual and olfactory variables to test. We here preferred to test, for correlation, all pairs of parameters separately.

## Results

### General overview on morphology

Females of the studied miltogrammine species varied greatly in body size (Table 1), with the smallest species (*T. heteroneura*) having an average head width roughly 50% smaller than the largest species (*M. germari*) (Table 1). SAT species and NON-SAT species had similar head widths (2.13 ± 0.12 mm vs. 1.89 ± 0.09 mm, respectively, Student’s t-test, *t* = 1.56, *df* = 17, *P* = 0.13). Similarly, we found the selected morphological variables used in this study displayed important variability among species.

**Table 1:** Mean ± Standard Error of the head width and the five morphological variables associated with the visual sensory system, calculated across individuals for each species.

Species	H_width_	E_area_		OM_area_		OM_number_	∆γ	OC_diameter_
*A. signata*	2297.6 ± 109.0	887826.7 ± 93027.5		341.2 ± 21.9		2585.2 ± 167.9	3.06 ± 0.10	55.7 ± 3.0
*A. seriemaculata*	1494.0 ± 68.4	395699.1 ± 27351.6		273.7 ± 14.0		1447.9 ± 72.6	4.08 ± 0.11	54.0 ± 3.4
*C. tabaniformis*	2394.1 ± 76.5	615394.8 ± 28390.5		413.3 ± 18.5		1489.9 ± 23.1	4.00 ± 0.03	66.3 ± 3.0
*E. persolla*	2153.3 ± 64.3	426282.4 ± 11766.2		285.9 ± 18.2		1502.6 ± 68.2	3.99 ± 0.09	61.8 ± 10.0
*M. argyrocephala*	2012.0 ± 34.4	441396.2 ± 13343.4		282.8 ± 14.0		1570.6 ± 38.8	3.90 ± 0.05	36.7 ± 0.4
*M. germari*	2559.0 ± 62.6	827724.4 ± 27060.3		327.5 ± 5.4		2527.5 ± 71.0	3.07 ± 0.04	72.7 ± 2.6
*M. punctata*	2421.0 ± 90.4	936436.3 ± 75090.6		356.0 ± 27.8		2635.3 ± 82.2	3.01 ± 0.05	78.0 ± 2.6
*M. turanica*	2194.0 ± 40.1	893903.0 ± 31581.7		345.6 ± 8.0		2589.9 ± 87.6	3.04 ± 0.05	61.0 ± 1.6
*P. fedtshenkoi*	1984.6 ± 35.2	397453.1 ± 9953.1		270.0 ± 9.3		1481.2 ± 58.1	4.02 ± 0.08	60.8 ± 2.1
*P. kocaki*	2049.0 ± 62.1	417403.2 ± 22927.2		294.0 ± 5.8		1416.9 ± 62.9	4.12 ± 0.09	67.8 ± 2.8
*P. pictipennis*	1900.1 ± 28.9	381743.4 ± 14846.8		276.6 ± 6.1		1381.8 ± 53.3	4.17 ± 0.08	43.3 ± 1.2
*P. melanura*	1867.6 ± 47.0	536610.9 ± 24617.6		241.7 ± 8.4		2219.5 ± 56.5	3.28 ± 0.04	64.1 ± 1.1
*S. albifrons*	2023.8 ± 59.3	584752.0 ± 30448.3		332.0 ± 13.9		1761.9 ± 62.1	3.69 ± 0.06	71.6 ± 6.6
*S. conica*	1598.3 ± 57.7	383844.8 ± 28873.1		252.7 ± 12.7		1510.5 ± 60.9	3.99 ± 0.09	66.1 ± 3.1
*S. tricuspis*	2464.8 ± 47.6	642024.6 ± 17713.1		300.3 ± 5.2		2140.1 ± 72.2	3.34 ± 0.06	92.7 ± 6.1
*S. claripennis*	2020.7 ± 65.3	457034.3 ± 36559.1		299.1 ± 9.5		1521.2 ± 95.6	3.99 ± 0.12	47.3 ± 1.5
*T. heteroneura*	1357.8 ± 76.1	252067.9 ± 16010.4		224.7 ± 7.8		1123.8 ± 66.2	4.63 ± 0.14	46.0 ± 3.0
*T. stictica*	1614.4 ± 32.3	306100.5 ± 10115.5		239.1 ± 7.4		1282.2 ± 30.8	4.32 ± 0.05	57.7 ± 2.9

All measures are in µm (linear) or µm^2^ (area).

*H* head, *E* eye, *OM* ommatidia, *OC* ocelli, ∆*γ* interommatidial angle.

**Figure 2:**
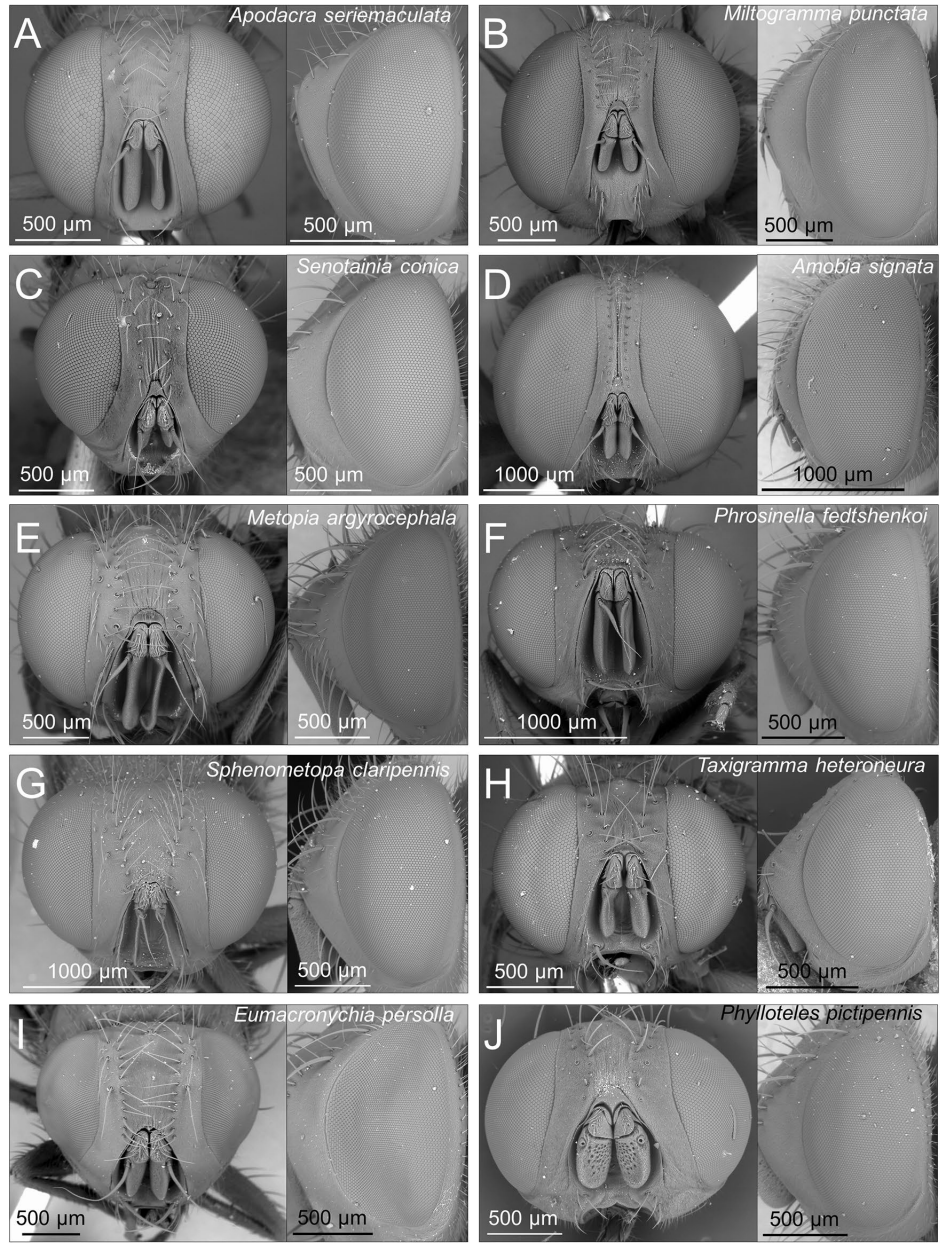
SEM pictures showing the variability of visual system in miltogrammine flies, as exemplified by a selection of the studied species. Head in frontal and lateral views are shown for each species. (**A**) *Apodacra seriemaculata*; (**B**) *Miltogramma punctata*; (**C**) *Senotainia conica*; (**D**) *Amobia signata*; (**E**) *Metopia argyrocephala*; (**F**) *Phrosinella fedtshenkoi*; (**G**) *Sphenometopa claripennis*; (**H**) *Taxigramma heteroneura*; (**I**) *Eumacronychia persolla*; (**J**) *Phylloteles pictipennis*. (**A**–**D**) are SAT species, (**E**–**J**) are NON-SAT species.

The general morphology of the visual system seems to differ to some extent among species, particularly in relation to the evident variability in eye size (Table 1, Fig. 2). Some species, such as *A. signata*, *M. punctata* and *S. conica* (all SAT species) have very large eyes covering large portions of the head when seen in frontal view, while other species, like *M. argyrocephala*, *P. pictipennis* and *T. heteroneura* (all NON-SAT species) have smaller eyes clearly covering a reduced portion of the head capsule (Fig. 2). Essentially all variables of the visual system appeared to have, on average, greater values in SAT than in NON-SAT species (Table 1). Eye area was 1.74 times greater in SAT than in NON-SAT species. Ommatidium number was also visibly greater in SAT species, which had 1.48 times larger ommatidia compared to NON-SAT species. Ommatidium area, instead, had a less pronounced variability being only 1.17 times greater in SAT than in NON-SAT species. Ocellar diameter was 1.29 times greater in SAT species.

**Figure 3:**
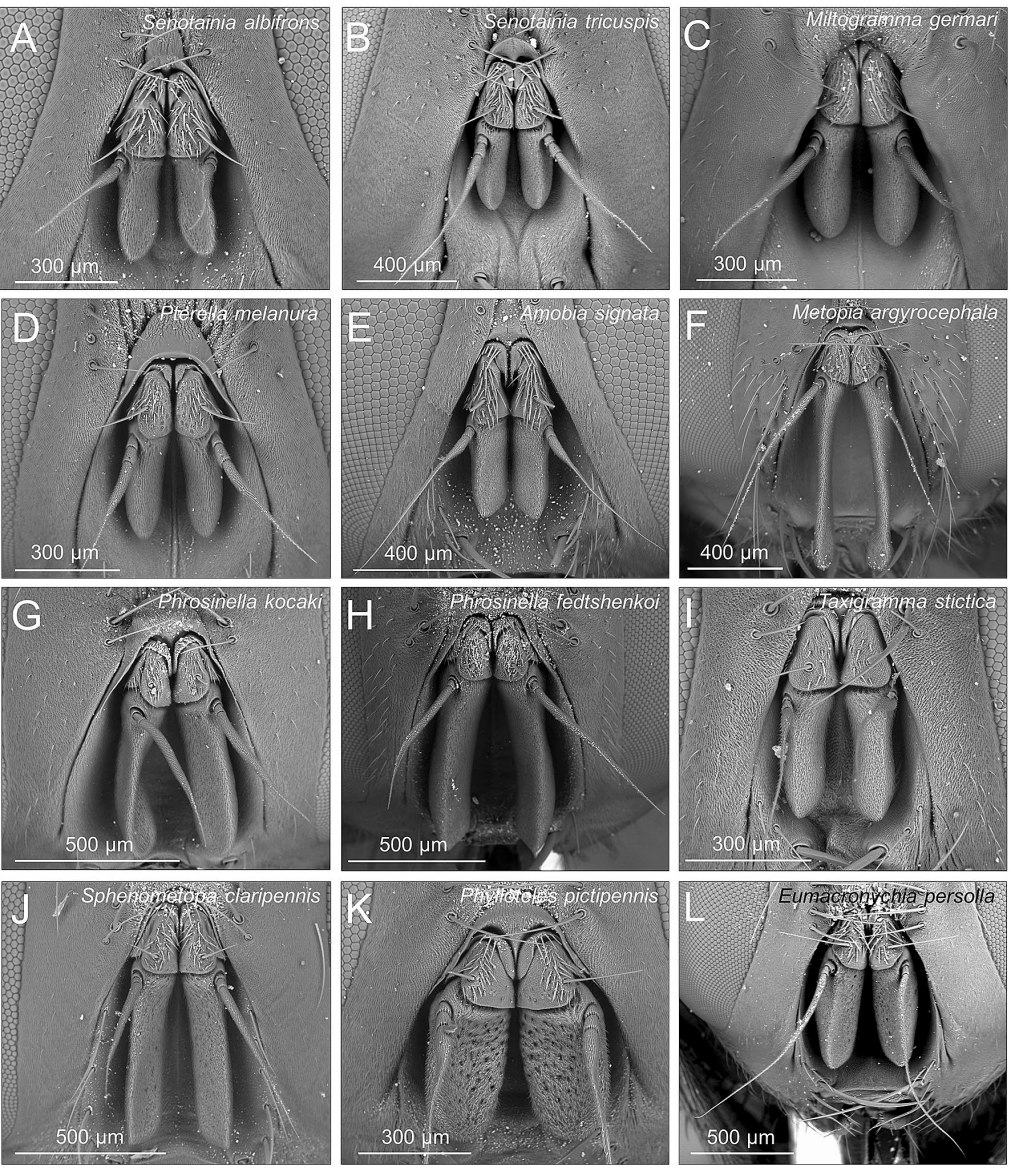
SEM pictures showing the variability of antenna in miltogrammine flies, as exemplified by a selection of the studied species. Head in frontal view is shown for each species. (**A**) *Senotainia albifrons*; (**B**) *Senotainia tricuspis*; (**C**) *Miltogramma germari*; (**D**) *Pterella melanura*; (**E**) *Amobia signata*; (**F**) *Metopia argyrocephala*; (**G**) *Phrosinella kocaki*; (**H**) *Phrosinella fedtshenkoi*; (**I**) *Taxigramma stictica*; (**J**) *Sphenometopa claripennis*; (**K**) *Phylloteles pictipennis*; (**L**) *Eumacronychia persolla*. (**A**–**E**) are SAT species, (**F**–**L**) are NON-SAT species.

**Figure 4:**
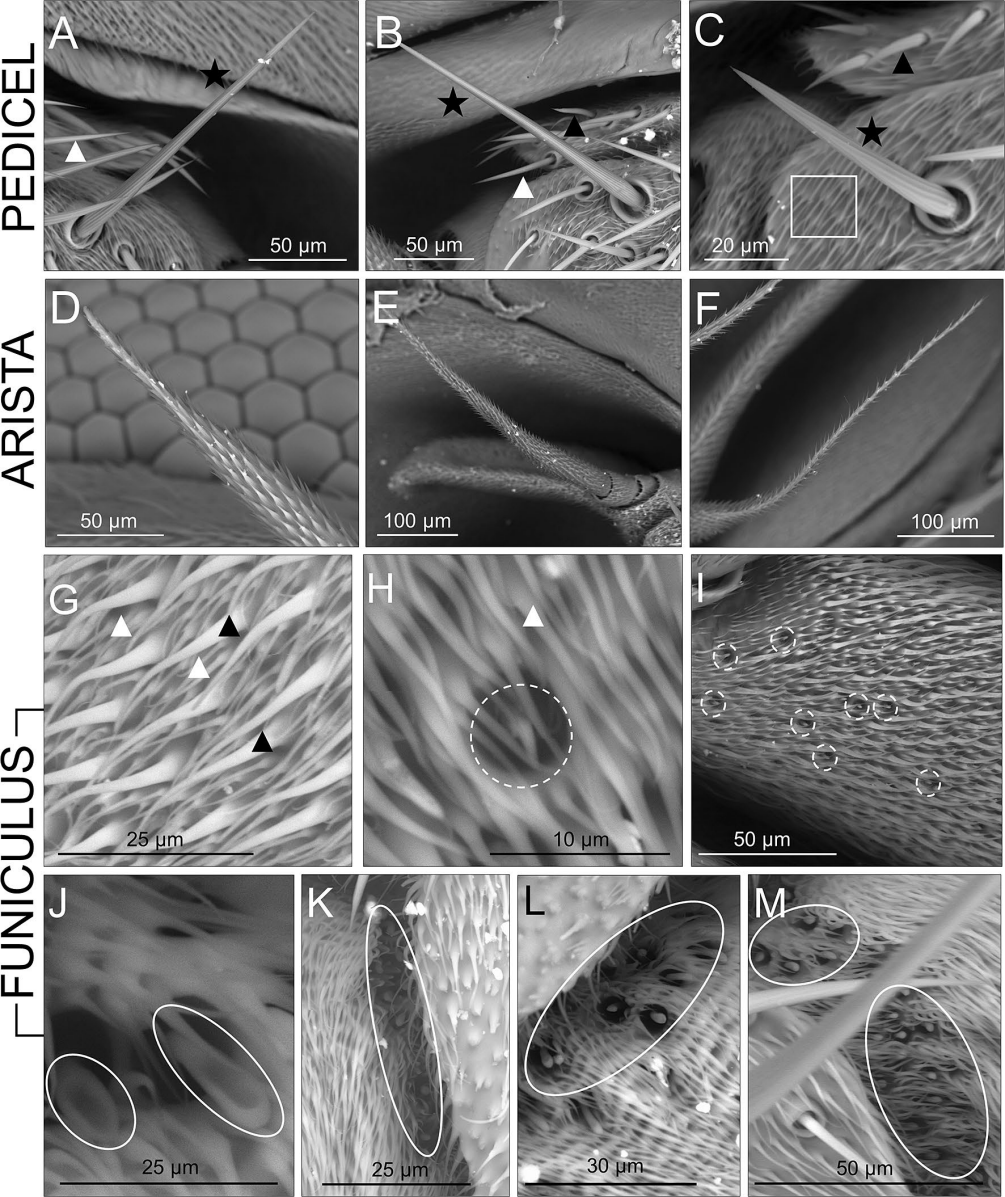
SEM pictures showing details of the sensillar types found on the antennae of the studied miltogrammine species. (**A**–**C**): Pedicel. (**A**), *Amobia signata* (SAT); (**B**), *Craticulina tabaniformis* (SAT); (**C**), *Taxigramma heteroneura* (NON-SAT). Black stars indicate very long chaetic sensilla (bristle, **B**), white triangles indicate the shorter narrow chaetic sensilla (NT), black triangles indicate the shorter thicker chaetic sensilla (TT). Note also the much denser and minute setae (EST) occuring all over the whole the pedicel (bold-line square in C). (**D**–**F**): Arista. (**D**), *Apodacra seriemaculata* (SAT); (**E**), *Miltogramma germari* (SAT); (**F**), *Metopia argyrocephala* (NON-SAT). The arista is finely covered by smooth chaetic sensilla (ST). G-M: Funiculus. (**G**) *Metopia argyrocephala* (NON-SAT); (**H**, **I**), *Phrosinella fedtshenkoi* (NON-SAT); (**J**), *Miltogramma punctata* (SAT); (**K**), *Phrosinella kocaki* (NON-SAT); (**L**) *Miltogramma turanica* (SAT); (**M**), *Phylloteles pictipennis* (NON-SAT). Black triangles indicate large smooth sensilla chaetica (LC), white triangles indicate small smooth sensilla chaetica (SC), dashed-line ovals include the rarer and sparse cone-shape sensilla basiconica (SB), and bold-line ovals indicate the “spoon-shaped” basiconic sensilla (SS), which seem to be concentrated in particular fields on the funiculus.

Despite its essentially similar structure, the antenna of the studied species varies in its morphology (Table 2, Fig. 3). The funiculus was quite large in some species such as *M. argyrocephala*, *P. fedtshenkoi* and *P. pictipennis* (all NON-SAT species) and reduced in other species such as *A. signata*, *P. melanura* and *S. tricuspis* (all SAT species) (Table 2, Fig. 3). On average, the funiculus was 1.24 longer in NON-SAT than in SAT species. The funiculus bears at least two types of very abundant smooth and small sensilla chaetica that differ strikingly in size (small chaetic sensilla (SC) and large chaetic sensilla (LC)), rare and sparse cone-shape sensilla basiconica (SB) and one type of “spoon-shaped” sensilla basiconica (SS) that seems to be concentrated in particular areas on the funiculus (Fig. 4G-M). In addition, olfactory pits occur on the funiculus with a roundish opening and surrounded by small chaetic sensilla, these pits are variable in number and size depending on the species (Fig. 4).

**Table 2:** Mean ± standard error of the four morphological variables associated with the antennal size, calculated across individuals for each species.

Species	F_length_	P_length_	A_length_	ANT_length_
*A. signata*	285.7 ± 17.8	232.4 ± 8.0	540.7 ± 46.1	1058.8 ± 60.5
*A. seriemaculata*	464.9 ± 34.7	173.0 ± 6.0	180.1 ± 27.7	818.0 ± 54.6
*C. tabaniformis*	498.9 ± 24.5	240.6 ± 5.0	358.6 ± 23.0	1241.3 ± 42.2
*E. persolla*	418.8 ± 70.5	255.3 ± 8.7	1005.8 ± 59.7	1679.7 ± 128.5
*M. argyrocephala*	625.4 ± 45.6	204.7 ± 6.5	761.8 ± 25.4	1571.5 ± 68.3
*M. germari*	339.3 ± 28.2	232.3 ± 4.2	449.7 ± 29.6	1021.3 ± 44.3
*M. punctata*	326.9 ± 23.6	221.9 ± 5.0	404.4 ± 10.2	953.1 ± 33.2
*M. turanica*	330.9 ± 13.6	211.9 ± 7.3	363.4 ± 11.2	901.8 ± 20.1
*P. fedtshenkoi*	470.1 ± 31.9	189.4 ± 4.2	544.3 ± 30.4	1203.8 ± 52.7
*P. kocaki*	471.3 ± 22.8	193.6 ± 6.7	487.1 ± 29.9	1152.0 ± 42.0
*P. pictipennis*	378.6 ± 7.9	188.6 ± 4.2	571.4 ± 28.0	1146.4 ± 27.1
*P. melanura*	271.3 ± 22.2	180.7 ± 7.0	285.9 ± 22.9	737.8 ± 27.4
*S. albifrons*	262.7 ± 20.6	191.7 ± 5.8	411.5 ± 34.8	865.8 ± 37.7
*S. conica*	199.3 ± 22.2	177.1 ± 7.0	395.3 ± 17.5	771.7 ± 40.8
*S. tricuspis*	335.5 ± 24.2	222.0 ± 2.7	576.0 ± 31.5	1133.5 ± 15.3
*S. claripennis*	423.9 ± 25.9	190.9 ± 4.8	521.3 ± 16.7	1127.7 ± 21.7
*T. heteroneura*	242.0 ± 15.5	175.5 ± 6.8	452.3 ± 31.0	870.2 ± 46.0
*T. stictica*	284.3 ± 7.2	187.6 ± 4.3	542.3 ± 31.8	1017.8 ± 31.7

All measures are in µm.

*F* funiculus, *P* pedicel, *A* arista, *ANT* antenna.

The pedicel was also quite variable in length (Table 2, Fig. 3) and was similar in length between SAT and NON-SAT species (ratio: 0.95). The pedicel bears a very long chaetic sensillum (the bristle, B), which is furrowed and sunk into a depressed base, and at least two types of shorter chaetic sensilla, which are overall similar in external morphology and resemble bristles except in size. The chaetic sensilla differ in thickness, with one type being narrow chaetic sensilla (NT) and the other type clearly thicker (thick chaetic sensilla, TT) (Fig. 4A-C). Additionally, extremely small setae (or microtrichia) (EST) occur in high densities on the pedicel.

The arista, which composes the flagellum together with the funiculus, was also visibly variable in length across species and was 1.54 times longer in NON-SAT than in SAT species. The arista is finely covered by small and smooth chaetic sensilla (ST) and seems to vary among species to some extent in both length and thickness, with some species having much thicker aristas, particularly towards the apex, than other species (Fig. 4D-F). Variability in the lengths of the funiculus, pedicel and arista lead to overall variability in the total length of antenna (Table 2), which overall resulted 1.24 times longer in NON-SAT species.

Overall the antennae of miltogrammine flies contain nine types of sensilla: bristle (B) (on the pedicel), narrow chaetic sensilla (NT), thick chaetic sensilla (TT), microtrichia (EST) (on the pedicel), smooth chaetic sensilla (ST) (on the arista) and small chaetic sensilla (SC), large chaetic sensilla (LC), cone-shape sensilla basiconica (SB) and “spoon-shaped” sensilla basiconica (SS) (on the funiculus). All these sensillar types occurred in all studied species.

### Comparative analysis and evolution of morphology

**Figure 5:**
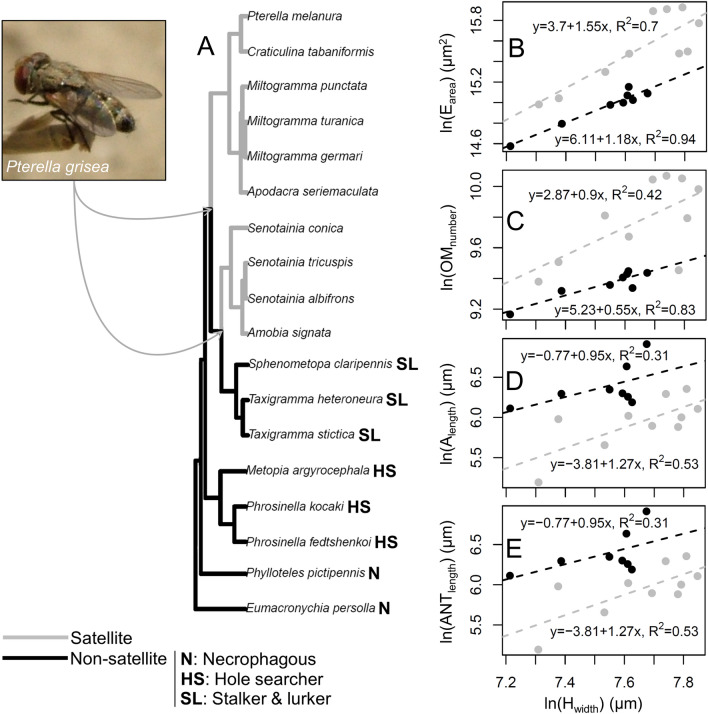
(**A**) Phylogenetic hypothesis used in phylogenetic comparative analyses with maximum parsimony reconstruction of satellite (grey) and non-satellite (black) behaviour. Host-finding strategy is reported for each species. Picture shows a female of a satellite fly species (*Pterella grisea*) perching on a small plant stick in the vicinity of a nest of its host species (the digger wasp *Cerceris rubida*) (picture taken nearby Alberese, Grosseto Province, Italy). Arrows connect the picture to the two clades of SAT species. (**B**–**E**), Charts depicting non-phylogenetic relationships (dashed lines) between head width and two visual (**B**, **C**) and two olfactory (**D**, **E**) traits. H_width_ = head width, E_area_ = eye area, OM_number_ = ommatidia number, A_length_ = arista length, ANT_length_ = antenna length. Gray circles and lines represent character values and relationships for SAT species, while black represents NON-SAT species. The equation and R^2^ are given for each relationship. All measurements are on the logarithmic scale.

The phylogenetic tree showed that satellite flight strategy is derived in Miltogramminae (Fig. 5A). All hole searchers and necrophagous species had non-satellite flight strategies as their ancestral state, with necrophagy being the ancestral strategy in the whole subfamily (Fig. 5A). On the other hand, satellite flight strategy was ancestral to a large group of species including all SAT species as well as stalkers and lurkers, implying that the latter have experienced a secondary loss of this peculiar behaviour (Fig. 5A). There was no difference between analyses with alternative maximum parsimony reconstructions of host-finding behavior.

Our statistical analysis revealed that most of the variables related to the visual system vary with host-funding strategy, as well as with body size. Eye area and ommatidium number are influenced by both host-finding behavior and by body size (R^2^ = 0.87), with SAT species having larger eyes and more ommatidia than NON-SAT species after controlling for head width (Fig. 5B, C, Table 3). In the case of these two traits, the model with head width and flight behaviour had the lowest AICc values (− 4.38 and − 3.19) and explain 87% and 71% of the variation, respectively. In both cases t_1/2_ were very short (0.00% and 0.04% of tree length) indicating almost instantaneous adaptation of these characters to flight behaviour and body size (Table 3). Taking into account that the overall phylogenetic effect for eye area and ommatidium number measured by fitting a model with only the intercept was moderate (13% and 17% of tree length) while the support region of t_1/2_ did not include 0 (Table 3), this significant drop in t_1/2_ indicated an absence of phylogenetic inertia in these eye traits. The allometric relationship among head width and both eye traits remained strong during the evolution of both non-satellite and satellite behaviours, while selection changed mainly the intercept, preferring bigger eyes and higher number of ommatidia in satellite species at similar body size in comparison with non-satellite ones (Fig. 5B, C). Consequently, smaller species had lower interommatidial angles (y = − 0.001x + 5.87, R^2^ = 0.52), and similarly SAT species had lower interommatidial angles than NON-SAT species (3.46 ± 0.14 vs. 4.14 ± 0.08) (Student’s t-test: *t* = 3.96, *df* = 17, *P* = 0.001).

**Table 3:** Phylogenetic comparative analyses of the evolution of visual system characters in the satellite and the non-satellite species. For each model, we show the maximum-likelihood estimates of phylogenetic half-life, t_1/2_, in units of tree length (total tree length = 1) with its 2LogL support region, the stationary variance, *v*_y_, GLS estimates of the slope of the optimal and evolutionary regressions with standard errors (SE). Phylogenetically corrected R^2^, in percentage, represents model fit. The best model from four tested for each response variable is chosen based on the best corrected Akaike Information Criterion (AICc) score and is given in bold. Models with Δ < 4 where Δ measures the information loss (or distance) of each model (AICc_i_) in comparison with the best one having the lowest AICc value (AICc_min_) according to equation Δ_i_ = AICc_i_ − AICc_min_, are considered equally supported and are denoted by asterisk. When a “–” is entered for the predictor variable, only an intercept is included in the model. In this cases, the phylogenetic half-life is a measure of the overall phylogenetic effect on the response variable. H = head, E = eye, OM = ommatidia, OC = ocelli, B_(0,1)_ = binary trait describing host-finding strategy (SAT vs. NON-SAT).

Response	Predictors	t_1/2_	*v*_y_	Optimal slopes (± SE)	Evolutionary slopes (± SE)	R^2^ (%)	AICc
E_area_	–	0.20 (0.07–1.09)	0.13	–	–	–	18.40
	H_width_	0.14 (0.00–∞)	0.03	1.81 ± 0.38	1.46 ± 0.30	56	8.66
	B_(0,1)_	0.08 (0.00–0.29)	0.07	–	–	43	14.10
	B_(0,1)_ + H_width_	0.00 (0.00–0.09)	0.02	1.42 ± 0.20	1.42 ± 0.20	87	**− 4.38**
OC_diameter_	–	0.09 (0.00–0.52)	0.05	–	–	–	2.85*
	H_width_	0.10 (0.00–∞)	0.03	0.69 ± 0.30	0.59 ± 0.26	22	1.84*
	B_(0,1)_	0.02 (0.00–0.18)	0.03	–	–	36	**− 0.84**
	B_(0,1)_ + H_width_	0.03 (0.00–0.36)	0.03	0.43 ± 0.24	0.41 ± 0.23	44	0.25*
OM_number_	–	0.17 (0.05–0.78)	0.07	–	–	–	7.83
	H_width_	0.13 (0.00–1.44)	0.03	1.04 ± 0.34	0.84 ± 0.27	35	4.15
	B_(0,1)_	0.06 (0.00–0.22)	0.04	–	–	47	2.27
	B_(0,1)_ + H_width_	0.04 (0.00–0.15)	0.02	0.80 ± 0.22	0.76 ± 0.21	71	**− 3.19**
OM_area_	–	0.09 (0.00–0.34)	0.02	–	–	–	− 10.40
	H_width_	0.00 (0.00–0.08)	0.01	0.70 ± 0.13	0.70 ± 0.13	64	**− 23.70**
	B_(0,1)_	0.04 (0.00–0.22)	0.02	–	–	21	− 10.40
	B_(0,1)_ + H_width_	0.00 (0.00–0.06)	0.01	0.63 ± 0.12	0.63 ± 0.12	70	− 22.90*

The results for two remaining visual characters, ocelli diameter and ommatidia area, were less conclusive (Table 3). Two of the four tested models, head width and head width + biology (B_(0,1)_), were equally supported for ommatidium area, suggesting the influence of both predictors on the evolution of this trait, although body size had a stronger effect (Supplementary Fig. S1A, Table 3). In the case of ocellar diameter, all models were within Δ < 4, although ocellar diameter seems much less constrained by body size than eye traits and the best model according to AICc criterion is that with host-finding behavior as a predictor (Supplementary Figure S1B, Table 3). Despite, on average, ocellar diameter being larger in SAT species (Student’s *t*-test: *t* = 2.94, *df* = 16, *P* = 0.009), models may indicate a weaker effect of head width and flight behaviour on the evolution of this visual character than on the other studied traits.

Our statistical analysis revealed that the size of the different parts of the antenna, as well as total antennal size, largely depended on body size (Fig. 5D, E, Supplementary Fig. S1C–F, Table 4), though antennal length and arista length were also affected by biology and follow the same pattern as eye area, with the models with host-finding behavior × head width as predictor variables explaining 70–75% of variance (Fig. 5D,E, Table 4). In the case of arista length, the phylogenetic inertia was strongly indicated by t_1/2_ = 0.67 and by differences between optimal and evolutionary slopes (3.43 ± 0.67 vs. 1.29 ± 0.25). Antennal length, on the other hand, showed almost instantaneous adaptation to flight behaviour and body size (t_1/2_ = 0.00) and identical slopes for optimal and evolutionary regression (0.82 ± 0.17). The non-phylogenetic regressions (Fig. 5) suggest a reverse pattern compared to visual system. Here, although the strong relationship of body size with both arista length and antennal length is sustained during the evolution of non-satellite and satellite behaviour, selection prefers longer arista and antenna in NON-SAT species (Fig. 5D,E, Table 4).

**Table 4:** Phylogenetic comparative analyses of the evolution of antennal size in the satellite and the non-satellite species. For each model, we show the maximum-likelihood estimates of phylogenetic half-life, t_1/2_, in units of tree length (total tree length = 1) with its 2LogL support region, the stationary variance, *v*_y_, GLS estimates of the slope of the optimal and evolutionary regressions with standard errors (SE).

Response	Predictors	t_1/2_	*v*_y_	Optimal slopes (± SE)	Evolutionary slopes (± SE)	R^2^ (%)	AICc
A_length_	–	0.26 (0.00–∞)	0.15	–	–	–	19.80
	H_width_	170.03 (2.88–∞)	6.59	602.58	1.23	56	9.21
	B_(0,1)_	0.10 (0.00–0.51)	0.08	–	–	35	16.30
	B_(0,1)_ + H_width_	0.67 (0.17–∞)	0.00	3.43 ± 0.67	1.29 ± 0.25	75	**2.66**
F_length_	–	0.28 (0.00–∞)	0.01	–	–	–	**11.80**
	H_width_	0.28 (0.00–∞)	0.08	0.80 ± 0.52	0.50 ± 0.32	12	12.90*
	B_(0,1)_	0.19 (0.00–∞)	0.08	–	–	13	12.90*
	B + H_width_	0.09 (0.00–1.00)	0.05	0.83 ± 0.38	0.72 ± 0.33	33	13.00*
P_length_	–	0.04 (0.00–0.27)	0.01	–	–	–	− 19.00
	H_width_	0.14 (0.00–0.90)	0.00	0.72 ± 0.11	0.58 ± 0.01	72	**− 37.10**
	B_(0,1)_	0.00 (0.00–0.23)	0.01	–	–	5	− 16.40
	B_(0,1)_ + H_width_	0.13 (0.00–0.80)	0.00	0.71 ± 0.11	0.58 ± 0.01	72	− 33.30*
ANT_length_	–	0.13 (0.00–2.12)	0.05	–	–	–	3.69
	H_width_	0.46 (0.03–∞)	0.03	1.38 ± 0.44	0.67 ± 0.21	35	− 0.31
	B_(0,1)_	0.00 (0.00–0.35)	0.03	–	–	31	0.41
	B_(0,1)_ + H_width_	0.00 (0.00–0.17)	0.01	0.82 ± 0.17	0.82 ± 0.17	70	**− 10.30**

Phylogenetically corrected R^2^, in percentage, represents model fit. The best model from four tested for each response variable is chosen based on the best corrected Akaike Information Criterion (AICc) score and is given in bold. Models with Δ < 4 where Δ measures the information loss (or distance) of each model (AICc_i_) in comparison with the best one having the lowest AICc value (AICc_min_) according to equation Δ_i_ = AICc_i_ – AICc_min_, are considered equally supported and are denoted by asterisk. When a “–” is entered for the predictor variable, only an intercept is included in the model. In this cases, the phylogenetic half-life is a measure of the overall phylogenetic effect on the response variable. H = head, F = funiculus, P = pedicel, A = arista, ANT = antenna, B_(0,1)_ = binary trait describing host-finding strategy (SAT vs. NON-SAT).

AICc values for pedicel length analysis showed support for more than one model, with this trait preferring both models with only head width and head width + biology (B_(0,1)_) as a predictor, suggesting a stronger influence of body size on its evolution (Table 4). Hence, the variance explained by head width is quite high in pedicel length (Supplementary Figure S1D). Pedicel length immediately responds to changes in body size without any detectable lag (t_1/2_ is close to 0) (Table 4). All models tested for funiculus length were supported equally by AICc values, precluding any sensible conclusions, and this trait seems largely dependent on phylogeny (Supplementary Fig. S1F, Table 4). While host-finding behavior does not seem to be a factor influencing the evolution of funiculus length, it is interesting to note that this failed relationship seems to be dependent on only two species. In fact, the ratio of funiculus length to head width is consistently lower in SAT species (12.5–15.1) than in NON-SAT species (17.6–31.1), with the exception of the SAT species *A. seriemaculata* and *C. tabaniformis*, which have a large funiculus relative to their body size (31.1and 20.8, respectively).

**Figure 6:**
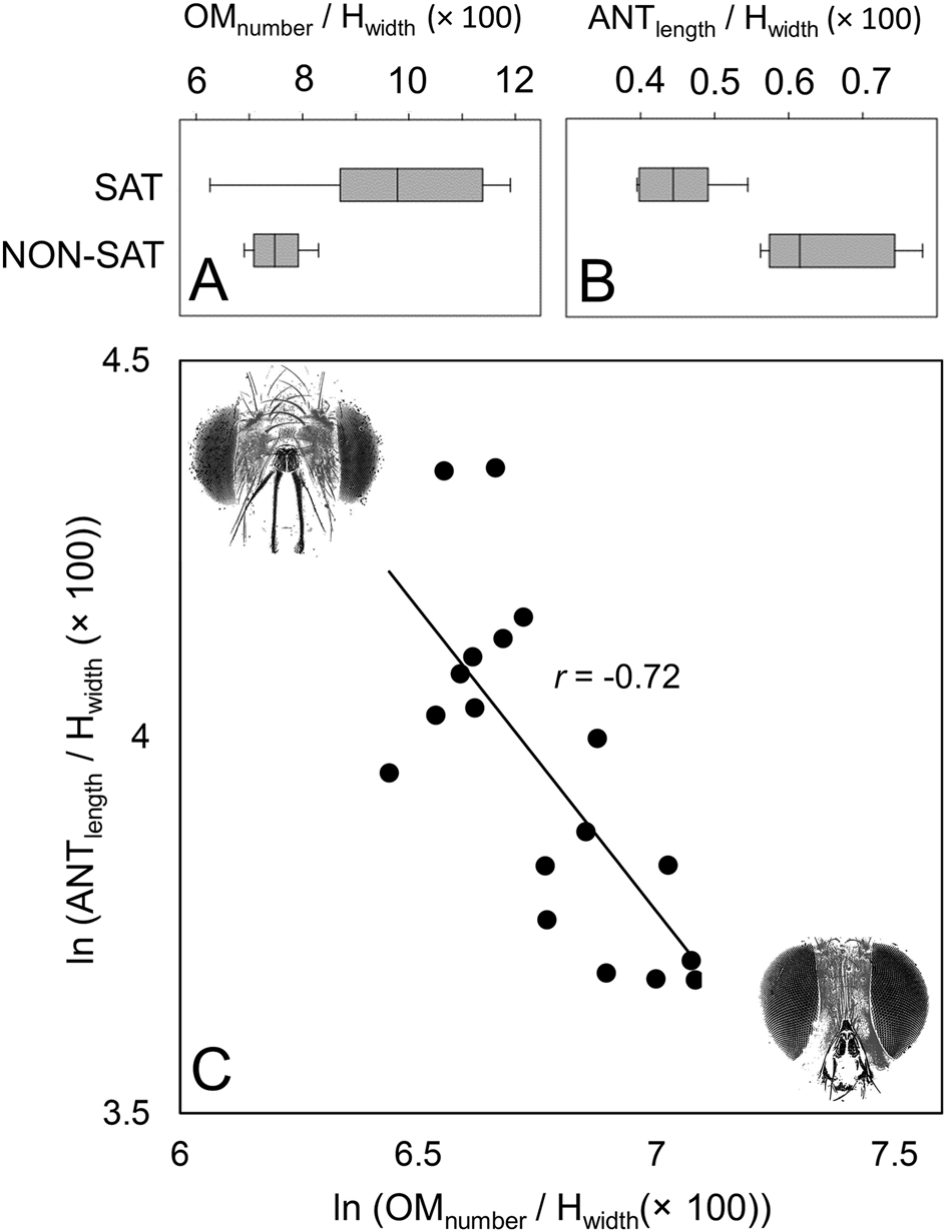
Relationship of host-foraging strategy (SAT and NON-SAT) with ommatidia number (OM_number_) (**A**) and antennal length (ANT_length_) (**B**), relative to H_width_. (**C**) Relationship between ln-transformed values of OM_number_ and ANT_length_, relative to H_width,_ across the studied species. Pearson correlation’s r is shown. Modified (to highlight eyes and antennae) pictures represent, in the upper left and in the lower right, respectively, the typical morphology of a NON-SAT species (smaller eyes and larger antennae, *M. argyrocephala*) and the typical morphology of a SAT species (larger eyes and smaller antennae, *P. melanura*).

SAT and NON-SAT species seem to have opposite trends in the investment of eyes and antennae. Indeed, while SAT species have an overall improved visual system (ommatidium number, which is strongly correlated to eye area, see above) (Fig. 6A), NON-SAT species present an overall longer antenna (Fig. 6B). This led to a number of significant inverse correlations between several pairs of visual system-related traits and antennal system-related traits (relative to body size) across species. In particular, antennal length, arista length and funiculus length (the latter to a lesser strength) decreased with increasing ommatidium number and eye area (− 0.75 < r < − 0.49, 0.0003 < *p* < 0.04) (Fig. 6C).

## Discussion

In this study, we hypothesized that visual system of miltogrammine flies evolved in response to the host-searching strategy, which is associated with strikingly different behaviours (i.e. employing or not employing satellite flights to reach the host nests). More specifically, we hypothesized that fly species which employ satellite flights to reach their host nests evolved an improved visual system, compared to species which do not perform such behaviours. Our hypothesis was well justified, as insect sensory systems are known to be strongly dependent on selective pressures^2,4^, leading to common links between components of the sensory system and different life-history or behavioural traits^5–7^. Our results largely reveal that such a link also exists in miltogrammine flies.

We have found that fly species which perform satellite flights to reach the host nests possess larger compound eyes compared to species which do not perform this specific behaviour. This could be related to the fact that increased eye size allows for improvement in visual quality^24^. Furthermore, depending on the interaction between ommatidia size and number, either light capture (larger but fewer ommatidia) or acuity (image resolution) (smaller but more numerous ommatidia) can be affected^21,22^. For example, crepuscular or nocturnal foraging (behaviours performed under low light availability) require greater visual acuity compared to diurnal foraging. Consequently, numerous studies have identified that insect groups with such temporal foraging window have some degree of selection for larger eyes^8,11,24,27^. The nocturnal bees *Megalopta genalis* Meade-Waldo and *Xylocopa tranquebarica* (Fabricius) have larger eyes and larger facets than the strictly day-active *Apis mellifera* L. and other *Xylocopa* spp., which is likely related to the former species needing to capture more light during their nocturnal behaviours^62–64^. Similar differences between nocturnal and diurnal species were found in wasps and ants^27,65–68^. In diurnal bee species, males which chase females or conspecific males in their territories also have enlarged eyes compared to species with non-territorial males^69^, which represents a similar situation to SAT species pursuing their flying hosts.

Interestingly, we have found a greater number of ommatidia, but not larger ommatidia, in SAT species compared to NON-SAT species. It seems that the basic unit of compound eye is evolutionary conserved. Selection to tune fly for satellite behaviour disproportionately changes the number of ommatidia and simultaneously eye area instead of area of ommatidia. Hence, it is likely that visual acuity, not light capture, is improved in the former group of flies. This is in general accordance with the observation that there is a trade-off between spatial resolution (number of ommatidia) and light sensitivity (facet diameters) in insects^22^, because increased resolution requires light to be sampled from a decreased region of space, subsequently reducing sensitivity^70^. Having a greater number of ommatidia makes sense for SAT species considering their complex flight behavior, in which flies precisely follow fast-flying hosts that often try to escape them (not always successfully) through elaborate, zig–zag evasive flights^40,41,71^. Our results also suggest allometric relationships with body size as a constraint for evolution of eye area and ommatidia number, i.e. even a small change in body size generates changes in these traits. However, it seems that this relationship is fine-tuned in SAT species by increasing the slope of the allometric line (satellite species have relatively larger eyes and more ommatidia than non-satellite species with comparable body sizes). Our rough estimation of the interommatidial angles further supports our hypothesis of improved vision in SAT species, given that SAT species had lower angles than NON-SAT species and it is known that smaller interommatidial angle equates to a greater distance at which objects can be resolved^22^. For this reason, lower interommatidial angles could favour SAT species while detecting and then chasing their flying hosts. Interestingly, even the stalker and lurker species in our sample showed a lower number of ommatidia and hence higher value of interommatidial angle compared to SAT species. This effectively suggests that selective pressure on visual system is caused by satellite flight behaviour and not only the visual detection of a nest-returning host. However, to substantiate this, other parameters are necessary to finely estimate visual acuity in insects, such as optical quality and rhabdom dimensions^21,22^, which could not be analysed in this study. It would also be interesting to complete a detailed study of the putative neurons that may respond selectively to small moving targets, as they have been previously found to play a role in detecting and pursuing such targets in other (male) flies^72,73^.

While the results of this study were inconclusive regarding the role of either SAT behaviour or body size on ocellar size, the ocelli were also larger, on average, in SAT species. Ocellar size had host-finding strategy as the best predictor, but our models do not contain clear predictors which would explain the differences in this trait or, perhaps, we don’t have enough data to support strongly any of the tested models. New analyses may confirm an evolutionary association between satellite behaviour and large ocelli, in turn supporting the fact that ocelli, by being tuned to finely capture light, have a role not only in light metering and determining the time of the day, but also in both maintaining stability during flight and motion perception^11,25,74^. Furthermore, neurological analyses suggest that ocelli, by mediating fast motor responses induced by sudden changes in light intensity, are stimulated in considerably less time than compound eyes, in order to elicit compensatory head movements^75^. Therefore, SAT species would favour larger ocelli, as chasing fast-moving hosts during satellite flight behaviour likely requires quick head movements to maintain a precise host-pursuing trajectory. Additionally, larger ocelli may be advantageous to escape risks associated with satellite flight behaviour, e.g., in case the persecuted host detects the satellite fly and attempt to attack it. Such a situation was reported in literature, though it does not seem to be common^41^, with evasive manoeuvres and/or nest abandonment more often observed as responses by the hosts^37,40,41,71^. We also cannot exclude that ocelli in miltogrammine flies are able to form low-resolution images. For example, in another calyptrate dipteran, although the lenses are highly underfocused, a poor quality astigmatic image can be formed at the retina for objects at certain spatial wavelengths^76^.

The external morphology of the three antennal segments of Miltogramminae is similar to that of other Sarcophagidae and Calyptratae in general (the brachyceran group of Diptera also including Sarcophagidae)^77^. However, detailed morphological studies based on SEM were rarely carried out on the antennal sensilla of Sarcophagidae^78–80^, and no previous study was published on Miltogramminae. Here, we provided the first description of antennal sensillar equipment in this subfamily, showing the occurrence of nine types of sensilla across all studied species, with chaetic sensilla of different sizes the most abundant. The nine sensillar types found in the studied species are similar to those found in other Sarcophagidae. The dense small setae or microtrichia covering both the pedicel and the funiculus, as well as the different types of chaetic sensilla, basiconic sensilla and olfactory pits did not differ greatly in shape, relative size nor position from those found in *Sarcophaga tibialis* Macquart^81^, *Wohlfahrtia nuba* Wiedemann^80^, *Sarcophaga babiyari* (Lehrer)^78^ and *Sarcophaga bullata* Parker^79^. The large bristle on the pedicel observed here closely resembled that also called “bristle” in other calyptrates^82,83^ and the sensillum called “chaetic sensillum type I” in *S. tibialis*^81^. Based on previous histological and/or physiological studies in insects, setae are not innervated, chaetic sensilla are thought to have either a mechano-tactile role or chemoreceptor role, likely dependant type, while basiconic sensilla have a chemoreceptor role^13,84,85^. Further studies are required to ascertain if there is a link between the morphology and function of sensilla in the Miltogramminae. In addition to the sensillar types found in our species, some authors have also reported further sensory structures in Sarcophagidae. For example, clusters of “setiferous plaques” (raised circular rims with a bulbous seta at its centre) have been described in the distal region of the pedicel in various species^78,81,86^. We did not observe similar structures in our sample, but we cannot exclude that they occur at locations in the pedicel that remained hidden in our SEM pictures.

Contrary to the visual system, the antennal system of Miltogramminae was less variable between SAT species and NON-SAT species, with an overall stronger role of body size on the variation of the studied morphological traits. It is well established that sensilla density increases with antennal size in insects, such that larger individuals were often suggested and on several occasions proven to have a greater sensitivity compared to smaller ones^4^. This could be also the case for larger species of Miltogramminae, but further studies are required to validate this hypothesis. However, the antennal system is complex, as two variables, antennal length and arista length, are influenced by both host-finding behavior and by body size, with SAT species having smaller antennae and smaller arista than NON-SAT species after controlling for H_width_. If it will be confirmed, as observed in other insects, that the number of sensilla increases with antennal size in miltogrammine flies, NON SAT species would perhaps possess an improved olfactory/gustatory as well as mechanosensory antennal system. Concerning the latter, in other dipterans some mechanoreceptor sensilla have been found to be used for the perception of air currents, which also helps in flight control^87^. Tactile hairs have also been reported to be an essential component of flight stabilisation and control in other insects, through the detection of wind by their deflection^18–20^. Interestingly, the arista length has been shown to be extremely important in flight in *Drosophila*, where incident wind causes arista deflection, and similarly arista vibration at the frequency of the nearby flapping wings^88^. However, in our analysis the arista was smaller in SAT species. This result may suggest that either the arista’s mechanoreceptors are not necessarily required in large number for performing satellite flights or that the role of the arista differ between *Drosophila* and Miltogramminae. In Diptera, fast mechanosensory feedback is provided by the halteres (their strongly modified hindwings) and is crucial for the control of rapid flight manoeuvres, while vision controls manoeuvres in lower temporal frequency bands^89^. Hence, miltogrammine flies may rely more on halters, and not on antennal mechanoreceptors, in conjunction to eyes, while performing satellite flights. In any case, only further analyses can elucidate if SAT and NON-SAT species really differ in mechanical and/or chemical sensitivity.

Despite our results for the antennal system, we found interesting correlations between different eye- and antenna-related variables that suggests an inverse resource allocation between these two structures in Miltogramminae. These contrasting patterns seem to stem from a theoretically restricted resource allocation between the two organs, both linked to a single imaginal disc (the resource), during larval development^43^. In our study it was not possible to determine if such inverse correlation in these structures’ size corresponds to an inverse allocation in vision and olfaction/taste and/or mechanoreception, since the specific functions of the different sensillar types remain unknown. A trade-off between vision and olfaction was reported in other insect species, especially while analysing the relative size of different parts of the brain^14,15,90^. A recent study in *Drosophila* demonstrated that such trade-off can be also detected while observing the peripheral sensory systems, and that this trade-off is linked with navigation ability: when only olfactory stimuli are tested, larger-antennae species navigate better, while when visual stimuli only are tested, larger-eyed species performed better^12^. It will be interesting to perform similar tests for the Miltogramminae in the future.

It is also important to highlight that our results are based on the phylogenetic reconstruction that we have used. Consequently, evolutionary trends of sensory equipment may be affected by alternative phylogenetic scenarios. Indeed, while the ancestral state of necrophagy and the derived state of SAT behaviour depicted in our tree are the same as those revealed by Piwczyński et al.^30^, Buenaventura et al.^51^ and Yan et al.^55^, the latter study resolved the position of stalkers and lurkers slightly differently. While we found that SAT behaviour is ancestral to a clade including all SAT species as well as stalkers and lurkers (implying their loss of SAT behaviour and consequent change in morphology), Yan et al.^55^ reported that the clade of stalkers and lurkers is sister to all SAT taxa, implying no evolutionary loss of SAT behavior and hence no change from the ancestral (non-SAT) morphology. It should be noted that the latter study used markedly lower taxon sampling, which may be one possible reason behind this difference. New evolutionary studies are needed to clarify this point. Furthermore, it would be useful to add male individuals in future research on morphological evolution in this fly subfamily, since one may expect that the differences found here between females of SAT and NON-SAT species may be less pronounced in males, which do not perform satellite behaviours. Nevertheless, differences in males could appear across species depending on variation in other life-history traits, such as their mating tactic (e.g. territorial vs. non-territorial behavior).

## Supplementary Information

Supplementary Information.
