# Supplementary material for: Host-trailing satellite flight behaviour is associated with greater investment in peripheral visual sensory system in miltogrammine flies
Source: Sci Rep. 2022 Feb 17;12:2773. doi: 10.1038/s41598-022-06704-8 (PMC8854417; doi:10.1038/s41598-022-06704-8)

Supplementary Information

Article: Host-trailing satellite flight behaviour is associated with greater investment in peripheral visual sensory system in miltogrammine flies

Authors: Carlo Polidori, Marcin Piwczynski, Federico Ronchetti, Krzysztof Szpila

Supplementary Table S1. List of the studied species, with the number of studied females, their origin and their resource-finding strategies. SAT: satellite flight strategy, NON-SAT: strategies which do not involve satellite flights (*N*: necrophagous, *HS*: hole searcher, *SL*: stalkers and lurkers). Cited sources include evidences for the known used strategy for the species, or in absence of clear data, for other species of the same genus. Personal observations on hosts by the authors of the present study are also cited (author's initials in brackets).

Species	*N* of individuals	Resource-finding strategy	References
Amobia signata (Meigen, 1824)	7	SAT	Fateryga (2012), personal observation (K.S.)
Apodacra seriemaculata Macquart, 1854	7	SAT	Grandi (1961), personal observation (K.S.)
Craticulina tabaniformis (Fabricius, 1805)	7	SAT	Krombein & Van Der Vecht (1987), personal observation (K.S.)
Eumacronychia persolla Reinhard, 1965	4	NON-SAT (*N*)	Szpila et al. (2010), Xu et al. (2018)
Metopia argyrocephala (Meigen, 1824)	7	NON-SAT (*HS*)	Pape (1987), Povolný & Verves (1997), personal observation (K.S.)
Miltogramma germari Meigen, 1824	6	SAT	Welch & Owens (2019), personal observation (K.S.)
Miltogramma punctata Meigen, 1824	7	SAT	Draber-Mońko (1969), personal observation (K.S.)
Miltogramma turanica Rohdendorf, 1935	7	SAT	Verves (1990), personal observation (K.S. & M.P.)
Phrosinella fedtshenkoi Rohdendorf, 1925	7	NON-SAT (*HS*)	Myartzeva (1972) Charykuliev and Myartzeva (1964), personal observation (K.S. & M.P.)
Phrosinella kocaki Verves & Khrokalo, 2017	7	NON-SAT (*HS*)	Personal observation (K.S. & M.P.)
Phylloteles pictipennis Loew, 1844	7	NON-SAT (*N*)	McGovan et al. (2001), Krohn (2007), Szpila and Pape (2007), Szpila et al. (2010),
Pterella melanura (Meigen, 1824)	7	SAT	Zimmermann et al. (2018), personal observation (K.S.)

Senotainia albifrons (Rondani, 1859)	6	SAT	Pape (1987), personal observation (K.S.)
Senotainia conica (Fallén, 1810)	7	SAT	Pape (1987), personal observation (K.S.)
Senotainia tricuspis (Meigen, 1838)	4	SAT	Giordani (1955), Pape (1987)
Sphenometopa claripennis (Villeneuve, 1933)	7	NON-SAT (*SL*)	Rohdendorf (1967), Povolný & Verves (1997), personal observation (K.S.)
Taxigramma heteroneura (Meigen, 1830)	6	NON-SAT (*SL*)	Pape (1987), Spofford & Kurczewski (1990), Kurczewski (2008), personal observation (K.S.)
Taxigramma stictica (Meigen, 1830)	7	NON-SAT (*SL*)	Spofford & Kurczewski (1990), Povolný & Verves (1997), Richet et al. (2013), personal observation (K.S.)

Literature cited

Charykuliev, D. M. & Myartzeva, S. N. On the biology of flies of the subfamily Miltogrammatinae (Diptera, Sarcophagidae). *Trans. Acad. Sci. Turk. SSR (Ser. Biol. Sci.)* **2**, 84-88 (1964).

Draber-Mońko, A. Ubersicht der in Polen vorkommenden *Mitlogramma*-Arten (Diptera, Sarcophagidae). *Polskie Pism. Ent.* **39**, 321-330 (1969).

Fateryga, A. V. Nesting of the wasp *Gymnomerus laevipes* (Hymenoptera, Vespidae) in the Crimea. *Vestnik Zoologii* **46**, 229–238 (2012) (in Russian).

Grandi, G. Studi di un entomologo sugli imenotteri aculeati. XXXI. *Boll. Entomol. Reg. I Super. Agr.* **25**, 1–659 (1961) (in Italian).

Krombein, K. V. & Van Der Vecht, J. Biosystematic studies of Ceylonese wasps, XVII: a revision of Sri Lankan and South Indian *Bembix* (Hymenoptera: Sphecoidea: Nyssonidae). *Smiths. Contrib. Zool.* **451**, 1-30 (1987).

Kurczewski, F. E. II. Nesting Behavior of *Tachysphex pechumani* (Hymenoptera: Crabronidae). *Northeast. Nat.* **15**, 33-66 (2008).

McGowan, A., Broderich, A. C., Deeming, J., Godley, B. J. & Han-Cock, E. G. Dipteran infestation of loggerhead (*Caretta caretta*) and green (*Chelonia mydas*) sea turtle nests in northern Cyprus. *J. Nat. Hist.* **35**, 573–581 (2001).

Myartzeva, S. N. The parasites and predators of the sphecoid wasps of Turkmenia. In: Tokgaev, T. & Myartzeva, S. N., editors. *Insects of Southern Turkmenia*. Ashkhabad, Turkmen SSR: Ylym. pp. 101-115 (1972).

Richet, R., Verves, Y. U. G. Whitmore, D. & Pape, T. Revision of *Taxigramma pseudaperta* Séguy, 1941 and comparisons with sympatric *T. multipunctata* (Rondani, 1859) (Diptera: Sarcophagidae). *Zootaxa* **2731**, 520-532 (2013).

Rohdendorf, B. B. The Palaearctic species of the genus *Sphenometopa* Townsend (Diptera, Sarcophagidae). *Entomol. Obozr*. **46**, 450–467 (1967) (in Russian with English summary).

Szpila, K. & Pape, T. Rediscovery, redescription and reclassification of *Beludzhia phylloteliptera* (Diptera: Sarcophagidae, Miltogramminae). *Eur. J. Entomol.* **104**, 119-137 (2007).

Szpila, K., Voss, J. G. & Pape, T. A new forensic indicator for buried bodies (Diptera, Sarcophagidae, Miltogramminae). *Med. Vet. Entomol*. **24**, 278–283 (2010).

Xu, W.-T., Zhang, D. & Pape, T. Biology of *Eumacronychia* Townsend, with a redescription of *E. persolla* Reinhard, 1965 (Diptera, Sarcophagidae). *ZooKeys* **783**, 55-65 (2018).

Welch, M. D. & Owens, N. W. The association of the kleptoparasitic satellite fly *Miltogramma germari* Meigen (Diptera, Sarcophagidae) with the pantaloons bee *Dasypoda hitripes* Fabricius (Hymenoptera, Melittidae). *Dipt. Digest* **26**, 209-218 (2019).

Verves, Y. A key to Sarcophagidae (Diptera) of Mongolia, Siberia and neighbouring territories. *Nasekomye Mongolii* **11**, 516-617 (1990) (in Russian).

Zimmermann, D., Wiesbauer, H., Schoder, S., Schuh, R., Sehnal, P. *et al.* Wiederentdeckung der sozialen Knotenwespe *Cerceris rubida* (Jurine, 1807) (Hymenoptera: Crabronidae) in Österreich, mit Angaben zur Biologie. *Beiträge zur Entomofaunistik* **19**, 111–120 (2018) (in German with English summary).

Supplementary Table S2. Origin of sample and morphological data for all studied individuals. H = head, E = eye, OM = ommatidia, OC = ocelli, Δγ = interommatidial angle, F = funiculus, P = pedicel, A = arista, ANT = antenna. Codes for countries: PL = Poland, HR = Croatia, ES = Spain, GR = Greece, TR = Turkey, IR = Iran, IL = Israel, UA = Ukraine, USA = United States of America. All measures are in µm. "bad" indicates a measure or a number that could be not clearly obtained.

Species	Ind.	Locality of collection	Year of coll.	H_{width}	E_{area}	OM_{area}	OM_{number}	$OC_{diameter}$	Δγ	F_{length}	P_{length}	A_{length}	ANT_{length}
Amobia signata	1	Slano (HR)	2003	2267.00	897406.11	283.71	3163.13	63.00	2.74	280.00	227.00	579.00	1086.00
Amobia signata	2	Iztuzu forest (TR)	2010	2502.00	1064390.92	349.59	3044.71	68.00	2.80	367.00	258.00	606.00	1231.00
Amobia signata	3	Akseki (TR)	2005	2311.00	811910.82	340.60	2383.74	56.00	3.16	309.00	223.00	467.00	999.00
Amobia signata	4	Kolymbari (GR)	2008	2202.00	766018.78	322.99	2371.65	50.00	3.17	217.00	215.00	484.00	916.00
Amobia signata	5	Zbocza Plutowskie (PL)	1997	2007.00	647736.55	305.84	2117.87	52.00	3.35	250.00	206.00	537.00	993.00
Amobia signata	6	Urša beach (HR)	2017	2811.00	1347049.01	463.02	2909.25	57.20	2.86	298.00	262.00	749.00	1309.00
Amobia signata	7	Zbocza Plutowskie (PL)	1998	1983.00	680275.03	322.99	2106.18	44.00	3.36	279.00	236.00	363.00	878.00
Apodacra seriemaculata	1	Pinet (ES)	2011	1432.00	292495.40	205.79	1421.35	50.00	4.09	513.00	150.00	141.00	804.00
Apodacra seriemaculata	2	Valencia (ES)	2011	1457.00	345918.73	270.30	1279.78	54.00	4.31	367.00	168.00	173.00	708.00
Apodacra seriemaculata	3	Pinet (ES)	2011	1574.00	411920.27	254.63	1617.72	61.50	3.84	458.00	182.00	260.00	900.00
Apodacra seriemaculata	4	Valencia (ES)	2011	1765.00	501208.37	305.84	1638.78	64.00	3.81	569.00	190.00	281.00	1040.00
Apodacra seriemaculata	5	Valencia (ES)	2011	1394.00	432015.72	317.22	1361.87	57.50	4.18	424.00	169.00	206.00	799.00
Apodacra seriemaculata	6	Pinet (ES)	2011	1206.00	340420.27	291.91	1166.18	37.00	4.52	347.00	159.00	84.00	590.00
Apodacra seriemaculata	7	Pinet (ES)	2011	1630.00	445914.93	270.30	1649.73	54.00	3.80	576.00	193.00	116.00	885.00
Craticulina tabaniformis	1	Valencia (ES)	2011	2483.00	637508.55	432.33	1474.58	73.00	4.02	548.00	243.00	371.00	1254.00
Craticulina tabaniformis	2	Valencia (ES)	2011	2405.00	627967.97	435.69	1441.31	70.80	4.07	528.00	246.00	416.00	1265.00
Craticulina tabaniformis	3	Pinet (ES)	2011	2093.00	497853.67	334.68	1487.55	51.00	4.00	379.00	229.00	352.00	1206.00
Craticulina tabaniformis	4	Pinet (ES)	2011	2345.00	615032.11	389.86	1577.56	60.00	3.89	505.00	243.00	335.00	1173.00
Craticulina tabaniformis	5	Arenal del Sol (ES)	2011	2316.00	591239.16	386.69	1528.99	71.00	3.95	466.00	244.00	397.00	1402.00
Craticulina tabaniformis	6	Valencia (ES)	2011	2764.00	749833.26	491.18	1526.58	71.00	3.95	578.00	260.00	402.00	1332.00
Craticulina tabaniformis	7	Arenal del Sol (ES)	2011	2353.00	588329.00	422.34	1393.03	67.00	4.13	488.00	219.00	237.00	1057.00
Eumacronychia persolla	1	Victorville (USA)	2004	2078.00	394954.30	259.80	1520.22	51.00	3.96	250.00	238.00	937.00	1425.00
Eumacronychia persolla	2	Victorville (USA)	2004	2011.00	424952.61	252.06	1685.89	40.00	3.76	355.00	245.00	911.00	1511.00
Eumacronychia persolla	3	Victorville (USA)	2004	2265.00	434101.86	303.03	1432.53	72.00	4.08	529.00	277.00	999.00	1805.00
Eumacronychia persolla	4	Victorville (USA)	2004	2259.00	451121.05	328.81	1371.98	84.00	4.17	541.00	261.00	1176.00	1978.00
Metopia argyrocephala	1	Toruń-instytut (PL)	2003	1974.00	427156.81	249.51	1711.97	35.00	3.73	447.00	185.00	655.00	1287.00
Metopia argyrocephala	2	Scorocice (PL)	2004	1975.00	463648.00	328.81	1410.08	37.00	4.11	585.00	218.00	819.00	1622.00
Metopia argyrocephala	3	Toruń-lotnisko (PL)	2002	1933.00	403208.11	262.40	1536.59	36.00	3.94	816.00	197.00	810.00	1823.00
Metopia argyrocephala	4	Toruń-lotnisko (PL)	2002	1987.00	465073.25	289.16	1608.34	bad	3.85	730.00	223.00	bad	bad

Metopia argyrocephala	5	Zbocza Plutowskie (PL)	2002	1990.00	405160.87	249.51	1623.81	37.00	3.83	545.00	186.00	777.00	1508.00
Metopia argyrocephala	6	Toruń-lotnisko (PL)	2002	2014.00	427160.58	262.40	1627.87	38.00	3.83	616.00	199.00	704.00	1519.00
Metopia argyrocephala	7	Toruń-lotnisko (PL)	2002	2211.00	498365.81	337.64	1476.04	37.00	4.02	639.00	225.00	806.00	1670.00
Miltogramma germari	1	Sierakowo (PL)	2011	2585.00	883283.57	331.74	2662.59	72.00	2.99	342.00	236.00	488.00	1066.00
Miltogramma germari	3	Samin (PL)	1996	2595.00	838087.43	308.67	2715.17	78.00	2.96	372.00	231.00	433.00	1036.00
Miltogramma germari	4	Biedrusko (PL)	2005	2301.00	717539.06	314.36	2282.55	74.00	3.23	391.00	222.00	338.00	951.00
Miltogramma germari	5	Toruń-lotnisko (PL)	2001	2671.00	843837.40	331.74	2543.68	61.00	3.06	238.00	221.00	486.00	945.00
Miltogramma germari	6	Gvypa (PL)	1998	2729.00	896466.62	343.59	2609.15	72.00	3.02	416.00	249.00	545.00	1210.00
Miltogramma germari	7	Rez. Gabowiek (PL)	2004	2473.00	787132.45	334.68	2351.89	79.00	3.18	277.00	235.00	408.00	920.00
Miltogramma punctata	1	Bydgoszcz-Fordon (PL)	2004	2739.00	1137615.17	393.05	2894.32	80.00	2.87	248.00	237.00	398.00	883.00
Miltogramma punctata	2	Waterloo (PL)	2007	2555.00	1115712.96	452.68	2464.71	84.00	3.11	290.00	226.00	403.00	919.00
Miltogramma punctata	3	Toruń-lotnisko (PL)	2002	2521.00	1017939.33	415.74	2448.51	85.00	3.12	399.00	240.00	434.00	1073.00
Miltogramma punctata	7	Kików (PL)	2004	2139.00	702721.01	283.71	2476.92	75.00	3.10	304.00	203.00	370.00	877.00
Miltogramma punctata	5	Zbocza Plutowskie (PL)	2004	2325.00	751235.66	305.84	2456.28	bad	3.11	344.00	219.00	422.00	985.00
Miltogramma punctata	6	Zbocza Plutowskie (PL)	2002	2099.00	730908.24	257.21	2841.70	66.00	2.90	286.00	214.00	371.00	871.00
Miltogramma punctata	4	Zbocza Plutowskie (PL)	2002	2569.00	1098921.89	383.52	2865.33	78.00	2.88	417.00	214.00	433.00	1064.00
Miltogramma turanica	1	Urša beach (HR)	2017	2097.00	797394.21	346.58	2300.75	61.00	3.22	354.00	197.00	314.00	865.00
Miltogramma turanica	2	Katoro (HR)	2016	2336.00	1016222.69	343.59	2957.70	64.00	2.84	332.00	243.00	378.00	953.00
Miltogramma turanica	3	Katoro (HR)	2016	2025.00	781281.06	317.22	2462.88	55.00	3.11	331.00	193.00	bad	bad
Miltogramma turanica	4	Katoro (HR)	2016	2221.00	887475.78	374.11	2372.22	62.00	3.17	367.00	216.00	bad	bad
Miltogramma turanica	5	Slano (HR)	2003	2254.00	949663.56	364.82	2603.12	57.00	3.02	323.00	210.00	389.00	922.00
Miltogramma turanica	6	Slano (HR)	2003	2260.00	935864.83	352.61	2654.13	68.00	3.00	352.00	230.00	359.00	941.00
Miltogramma turanica	7	Tatar (IR)	2016	2165.00	889418.89	320.10	2778.57	60.00	2.93	257.00	194.00	377.00	828.00
Phrosinella fedtshenkoi	1	Dehmalek (IR)	2017	2074.00	407615.33	291.91	1396.37	70.00	4.13	418.00	187.00	480.00	1085.00
Phrosinella fedtshenkoi	2	Daraq (IR)	2014	1951.00	403914.84	289.16	1396.84	51.30	4.13	405.00	188.00	571.00	1164.00
Phrosinella fedtshenkoi	3	Sarcheshmeh (IR)	2014	2068.00	442354.41	289.16	1529.77	62.00	3.95	639.00	199.00	658.00	1496.00
Phrosinella fedtshenkoi	4	Anduhjred (IR)	2015	1798.00	361517.31	283.71	1274.26	61.00	4.32	525.00	199.00	482.00	1206.00
Phrosinella fedtshenkoi	5	Daraq (IR)	2014	1991.00	403082.11	232.01	1737.36	62.00	3.70	454.00	198.00	470.00	1122.00
Phrosinella fedtshenkoi	6	Gohargaz (IR)	2017	2017.00	372117.95	259.80	1432.32	60.00	4.08	425.00	168.00	645.00	1238.00
Phrosinella fedtshenkoi	7	Darband II (IR)	2016	1993.00	391570.01	244.45	1601.87	59.00	3.86	425.00	187.00	504.00	1116.00
Phrosinella kocaki	1	Dehmalek (IR)	2017	2237.00	507954.42	322.99	1572.66	bad	3.89	580.00	223.00	483.00	1286.00
Phrosinella kocaki	2	Dehmalek (IR)	2017	2209.00	468073.91	297.45	1573.65	65.00	3.89	456.00	201.00	624.00	1281.00
Phrosinella kocaki	3	Dehmalek (IR)	2017	2181.00	428754.75	297.45	1441.46	82.00	4.06	496.00	209.00	534.00	1239.00
Phrosinella kocaki	4	Dehmalek (IR)	2017	1895.00	373964.27	275.62	1356.80	62.00	4.19	472.00	178.00	438.00	1088.00
Phrosinella kocaki	5	Dehmalek (IR)	2017	2068.00	440023.35	281.00	1565.92	62.00	3.90	394.00	182.00	490.00	1066.00
Phrosinella kocaki	6	Dehmalek (IR)	2017	1872.00	344331.46	289.16	1190.78	67.00	4.47	416.00	177.00	472.00	1065.00
Phrosinella kocaki	7	Darband II (IR)	2016	1881.00	358720.27	294.67	1217.36	68.50	4.42	485.00	185.00	369.00	1039.00
Phylloteles pictipennis	1	Toruń-lotnisko (PL)	2010	1792.00	322411.20	267.65	1204.59	43.20	4.45	349.00	183.00	bad	bad
Phylloteles pictipennis	2	Iztuzu beach (TR)	2010	1897.00	366861.98	270.30	1357.26	40.00	4.19	390.00	185.00	503.00	1078.00
Phylloteles pictipennis	3	Toruń-lotnisko (PL)	2010	1833.00	341818.52	275.62	1240.17	41.60	4.38	367.00	178.00	572.00	1117.00
Phylloteles pictipennis	4	Toruń-instytut (PL)	2010	1968.00	399784.25	303.03	1319.29	47.00	4.25	378.00	207.00	663.00	1248.00
Phylloteles pictipennis	5	Toruń-instytut (PL)	2010	1855.00	396694.18	262.40	1511.77	45.00	3.97	384.00	179.00	bad	bad

Phylloteles pictipennis	6	Sisangān (IR)	2014	1984.00	428674.37	294.67	1454.75	47.00	4.05	367.00	201.00	625.00	1193.00
Phylloteles pictipennis	7	Toruń-lotnisko (PL)	2010	1972.00	415959.33	262.40	1585.18	39.00	3.88	415.00	187.00	494.00	1096.00
Pterella melanura	1	Góra Żymierskiego (PL)	2006	1919.00	545810.89	257.21	2122.06	61.00	3.35	285.00	198.00	391.00	874.00
Pterella melanura	2	Ivanovka (UA)	2009	1695.00	462312.56	203.48	2272.01	67.00	3.24	263.00	165.00	256.00	684.00
Pterella melanura	3	Góra Żymierskiego (PL)	2005	1902.00	569792.96	249.51	2283.63	62.00	3.23	278.00	181.00	263.00	722.00
Pterella melanura	4	Góra Żymierskiego (PL)	2005	1751.00	466530.21	217.51	2144.86	62.00	3.33	192.00	162.00	305.00	659.00
Pterella melanura	5	Góra Żymierskiego (PL)	2005	2081.00	650888.40	262.40	2480.48	68.00	3.10	338.00	202.00	217.00	757.00
Pterella melanura	6	Góra Żymierskiego (PL)	2005	1865.00	511746.21	254.63	2009.76	66.00	3.44	202.00	160.00	332.00	694.00
Pterella melanura	7	Toruń-poligon (PL)	2002	1860.00	549195.11	246.97	2223.71	63.00	3.27	341.00	197.00	237.00	775.00
Senotainia albifrons	1	Arenal del Almoroxo (ES)	2011	1736.00	492092.40	314.36	1565.39	39.60	3.90	171.00	165.00	406.00	742.00
Senotainia albifrons	2	Arenal del Almoroxo (ES)	2011	2062.00	633625.94	358.69	1766.52	81.00	3.67	260.00	192.00	514.00	966.00
Senotainia albifrons	3	Iztuzu forest (TR)	2010	2118.00	650793.97	374.11	1739.57	71.00	3.70	314.00	203.00	318.00	835.00
Senotainia albifrons	4	Iztuzu beach (TR)	2010	2035.00	490363.12	294.67	1664.10	81.00	3.78	302.00	191.00	388.00	881.00
Senotainia albifrons	5	Görkbel (TR)	2010	2127.00	642798.82	352.61	1822.99	82.00	3.61	272.00	195.00	331.00	798.00
Senotainia albifrons	7	Toruń-lotnisko (PL)	2001	2065.00	598838.27	297.45	2013.27	75.00	3.44	257.00	204.00	512.00	973.00
Senotainia conica	1	Toruń-lotnisko (PL)	2009	1709.00	479353.73	289.16	1657.72	72.00	3.79	194.00	177.00	413.00	784.00
Senotainia conica	2	Kików (PL)	2004	1802.00	470133.75	297.45	1580.57	79.00	3.88	278.00	207.00	439.00	924.00
Senotainia conica	3	Dolina Narwi (PL)	2000	1632.00	369732.25	265.02	1395.10	56.00	4.13	264.00	195.00	387.00	846.00
Senotainia conica	4	Toruń-lotnisko (PL)	2001	1585.00	370427.76	252.06	1469.58	67.00	4.03	150.00	169.00	432.00	751.00
Senotainia conica	5	Toruń-lotnisko (PL)	2001	1567.00	362899.93	229.56	1580.85	68.00	3.88	196.00	176.00	428.00	800.00
Senotainia conica	6	Unistal (PL)	2001	1309.00	251075.66	205.79	1220.07	56.00	4.42	111.00	152.00	317.00	580.00
Senotainia conica	7	Góra Żymierskiego (PL)	2005	1584.00	383290.77	229.56	1669.68	65.00	3.78	202.00	164.00	351.00	717.00
Senotainia tricuspis	1	Knyazha Gora (UA)	2007	2413.00	615301.84	289.16	2127.87	80.10	3.35	402.00	217.00	495.00	1114.00
Senotainia tricuspis	2	Pekari (UA)	2007	2468.00	693394.03	297.45	2331.17	109.00	3.20	286.00	228.00	628.00	1142.00
Senotainia tricuspis	3	Pekari (UA)	2007	2381.00	622338.58	314.36	1979.71	88.60	3.47	330.00	218.00	557.00	1105.00
Senotainia tricuspis	4	Knyazha Gora (UA)	2007	2597.00	637064.32	300.23	2121.91	93.00	3.35	324.00	225.00	624.00	1173.00
Sphenometopa claripennis	1	Lotz Cisterns (IL)	2006	2046.00	464568.34	289.16	1606.59	47.50	3.85	462.00	186.00	468.00	1116.00
Sphenometopa claripennis	2	Lotz Cisterns (IL)	2006	1917.00	404920.66	300.23	1348.70	42.00	4.20	371.00	182.00	531.00	1084.00
Sphenometopa claripennis	3	Lotz Cisterns (IL)	2006	2175.00	551459.99	289.16	1907.08	47.00	3.53	463.00	202.00	bad	bad
Sphenometopa claripennis	4	Makhtesh Ramon (IL)	2004	2273.00	606669.74	337.64	1796.82	53.00	3.64	462.00	200.00	557.00	1219.00
Sphenometopa claripennis	5	Dehmalek (IR)	2015	2063.00	467097.92	328.81	1420.57	44.00	4.09	450.00	209.00	500.00	1159.00
Sphenometopa claripennis	6	Tehran (IR)	2016	1766.00	352760.55	275.62	1279.87	bad	4.31	470.00	174.00	488.00	1132.00
Sphenometopa claripennis	7	Tehran (IR)	2016	1905.00	351763.21	272.95	1288.73	50.00	4.30	289.00	183.00	584.00	1056.00
Taxigramma heteroneura	1	Jarki (PL)	2005	1502.00	294919.16	222.29	1326.72	50.00	4.24	249.00	192.00	471.00	912.00
Taxigramma heteroneura	2	Toruń-poligon (PL)	2006	1041.00	221724.51	244.45	907.05	36.00	5.12	210.00	151.00	341.00	702.00
Taxigramma heteroneura	3	Mala Nieszawka (PL)	2005	1462.00	273459.85	239.43	1142.12	41.00	4.57	294.00	186.00	bad	bad
Taxigramma heteroneura	4	Mala Nieszawka (PL)	2005	1428.00	231460.15	227.12	1019.09	42.00	4.83	269.00	171.00	487.00	927.00
Taxigramma heteroneura	5	Toruń Glinki (PL)	2001	1493.00	289932.92	224.70	1290.31	54.00	4.30	240.00	190.00	510.00	940.00
Taxigramma heteroneura	6	Toruń-lotnisko (PL)	2001	1221.00	200911.09	189.92	1057.87	53.00	4.75	190.00	163.00	bad	bad
Taxigramma stictica	1	Jarki (PL)	2005	1590.00	290395.13	239.43	1212.85	43.00	4.43	290.00	179.00	490.00	959.00
Taxigramma stictica	2	Kiślaki (PL)	2004	1635.00	308111.87	217.51	1416.53	60.00	4.10	255.00	197.00	545.00	997.00
Taxigramma stictica	3	Jarki (PL)	2005	1553.00	292812.30	252.06	1161.66	56.00	4.53	298.00	196.00	588.00	1082.00

Taxigramma stictica	4	Toruń-lotnisko (PL)	2004	1477.00	270914.18	212.78	1273.20	60.60	4.33	279.00	171.00	bad	bad
Taxigramma stictica	5	Toruń-lotnisko (PL)	2000	1642.00	327617.32	254.63	1286.64	67.00	4.30	297.00	190.00	592.00	1079.00
Taxigramma stictica	6	Toruń-lotnisko (PL)	2001	1747.00	352339.48	265.02	1329.47	62.00	4.23	264.00	201.00	636.00	1101.00
Taxigramma stictica	7	Czarny Brynsk (PL)	1997	1657.00	300513.70	232.01	1295.27	55.00	4.29	307.00	179.00	403.00	889.00

Supplementary Figure S1. Charts depicting non-phylogenetic relationships (dashed lines) between head width and two visual (A, B) and two olfactory (C-D) traits. H_{width} = head width, $OC_{diameter}$ = ocellar diameter, OM_{area} = ommatidium area, P_{length} = pedicel length, F_{length} = funiculus length. Gray circles and lines represent character values and relationships for SAT species, while black for NON-SAT species. The equation and R^2 are given for each relationship. All measurements are on the logarithmic scale.

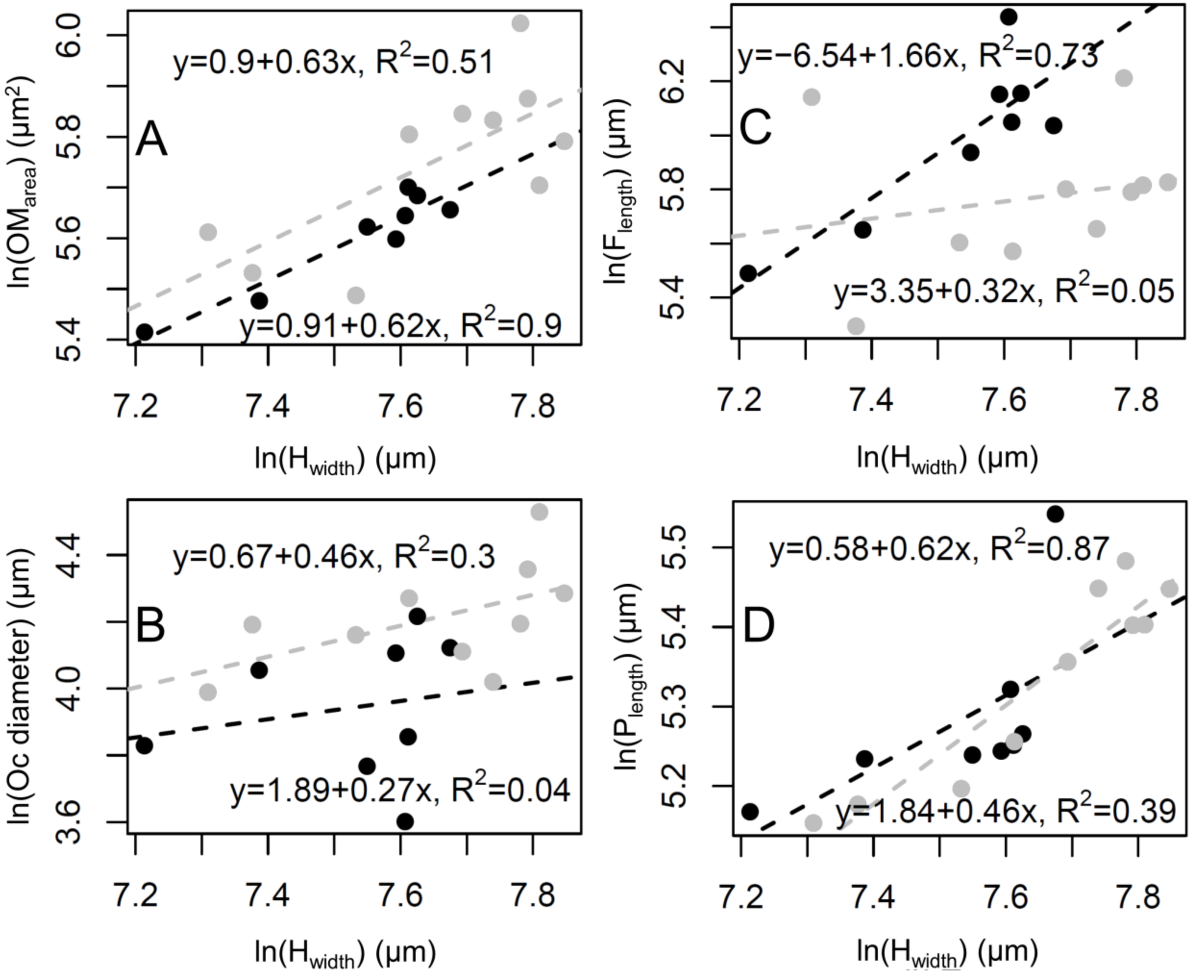

Supplement: Supplementary file 1 — Supplementary Information. [file 41598_2022_6704_MOESM1_ESM.pdf]
